# SIRT4-Mediated Deacetylation of PRDX3 Attenuates Liver Ischemia Reperfusion Injury by Suppressing Ferroptosis

**DOI:** 10.7150/ijbs.114510

**Published:** 2025-07-11

**Authors:** Qiwen Yu, Dongjing Yang, Binli Ran, Jie Pan, Yaodong Song, Mengwei Cui, Qianqian He, Chaopeng Mei, Haifeng Wang, Huihui Li, Guanghui Li, Yinuo Meng, Fazhan Wang, Wenzhi Guo, Changju Zhu, Sanyang Chen

**Affiliations:** 1 Department of Emergency Medicine, the First Affiliated Hospital of Zhengzhou University, Zhengzhou, 450052, Henan, China.; 2 Henan Medical Key Laboratory of Emergency and Trauma Research, Zhengzhou, 450052, Henan, China.; 3 Henan Emergency and Trauma Medicine Engineering Research Center, Zhengzhou, 450052, Henan, China.; 4 Department of Hepatobiliary and Pancreatic Surgery, the First Affiliated Hospital of Zhengzhou University, Zhengzhou, 450052, Henan, China.; 5 Henan Key Laboratory of Digestive Organ Transplantation, Zhengzhou, 450052, Henan, China.; 6 Biomedical Sciences, King's College, London, WC2R 2LS, United Kingdom.; 7 Medical Research Center, the First Affiliated Hospital of Zhengzhou University, Zhengzhou, 450052, Henan, China.

**Keywords:** SIRT4, liver ischemia reperfusion injury, PRDX3, deacetylation, ferroptosis

## Abstract

Liver ischemia-reperfusion injury (LIRI) is an important cause of the clinical prognosis of liver transplantation. Despite Sirtuin 4 (SIRT4) is involved in various post-translational modifications, its role in LIRI is unclear. This research aimed to investigate the influence of SIRT4 on the pathogenesis of LIRI. To this end, SIRT4 knockout (KO) and liver-specific overexpression mice, as well as alpha mouse liver 12 (AML12) cells, were employed. We showed that SIRT4 expression was downregulated in mice with LIRI or AML12 cells exposed to hypoxia-reoxygenation (H/R) injury, as well as in the liver tissue of liver transplant patients. SIRT4 KO exacerbated liver injury and ferroptosis; conversely, liver-specific SIRT4 overexpression in mice produced the opposite results. Furthermore, the ferroptosis inhibitor ferrostatin-1 mitigated the exacerbation of liver injury and ferroptosis caused by SIRT4 KO. Mechanistically, SIRT4 interacted with peroxiredoxins 3 (PRDX3) and deacetylated it at lysine 92, leading to the inhibition of ferroptosis. Furthermore, the protective effect of SIRT4 on LIRI was dependent on PRDX3 deacetylation at lysine 92. Additionally, liver-targeted lipid nanoparticles (LNPs)-sirt4 mRNA alleviated LIRI and ferroptosis in mice. Taken together, our findings highlight the SIRT4-PRDX3 axis as a key regulator and potential therapeutic target for LIRI.

## Introduction

Liver ischemia-reperfusion injury (LIRI) is a pathophysiological process in which a series of complex cascade reactions are initiated when the liver tissue resumes blood flow after a period of ischemia, leading to further exacerbation of liver injury 1, 2. Clinically, LIRI often occurs during liver operations such as liver transplantation and hepatectomy 3. LIRI is closely associated with 10% of early acute liver transplant rejection and often causes a range of complications, including severe decline in liver function within a short period of time after surgery and disorders of the coagulation system, and can also lead to liver failure and even death in severe cases, which greatly affects the therapeutic effect of liver transplantation 3-5. Therefore, investigating the mechanism of LIRI and finding new therapeutic measures to reduce LIRI are essential.

Ferroptosis, a newly characterized form of cell death that is dependent on the presence of iron, has attracted significant interest 6; it is characterized by the excessive accumulation of lipid peroxides and reactive oxygen species (ROS), setting it apart from other regulated cell death mechanisms, such as apoptosis, necrosis, and autophagy, at both the morphological and molecular levels 7, 8. Ferroptosis has been reported to participate in the occurrence and development of multiple diseases 9. There is an increasing consensus that LIRI can induce ferroptosis in hepatocytes, contributing to liver injury 10, 11. Studies have shown that the ferroptosis inhibitor ferrostatin-1 can reduce LIRI 12. Furthermore, promoting ferroptosis may worsen LIRI 13, 14. Targeting ferroptosis represents a new way to treat LIRI.

Sirtuins, a family of deacetylases comprising seven members (SIRT1-SIRT7), are involved in a range of biological processes including cellular metabolism, hyperinflammation, and cell death 15-17. In particular, SIRT4 is predominantly found in the mitochondria and plays multiple roles through post-translational modifications in both physiological and pathological processes 18. The deacetylase activity of SIRT4 has significant implications for the structure and function of various proteins, playing a critical role in many diseases and influencing their onset and progression 19-21. SIRT4 has been implicated in the regulation of a variety of diseases, including liver disease, through the regulation of ferroptosis. For example, advanced glycosylation end product receptor 1 (AGER1) deletion downregulates SIRT4 expression, induces ferroptosis and promotes fibrosis progression in nonalcoholic steatohepatitis with type 2 diabetes 22. However, to our knowledge, as an important regulator of ferroptosis, no previous studies have explored the role of SIRT4 in LIRI.

The aim of this study was to investigate the effect of SIRT4 on LIRI, with a focus on its role in inhibiting ferroptosis. To test our hypothesis, a series of experiments were performed to investigate the involvement of SIRT4-mediated peroxiredoxin 3 (PRDX3) deacetylation in the inhibition of ferroptosis upon I/R exposure. These findings provide insight into the mechanisms of LIRI pathogenesis and open new avenues for targeted therapy in this patient population.

## Materials and Methods

### Animals

Wild type male C57BL/6 mice aged 6-8 weeks were purchased from Jinan Xinbainuo Biotechnology Co., Ltd. SIRT4 global knockout (KO) mice were generated by Cyagen Biosciences (Suzhou, China). A liver-targeted AAV8 system containing a green fluorescent protein (GFP) scramble control, SIRT4, PRDX3-wild type (WT), PRDX3-K92-R, or PRDX3-K92-Q (provided by OBiO Technology Co., Ltd., Shanghai, China) was delivered into the mice via the tail vein at a dose of 5×10^11^ vg (200 μL per mouse). The mice were raised in a specific pathogen-free environment with a 12-hour light/dark cycle. Water and food were available *ad libitum*. Before the experiment, the animals were allowed to acclimate to the environment for one week. All experimental protocols followed the National Institutes of Health Guide for the Care and Use of Laboratory Animals and the ARRIVE guidelines, and were approved by the Ethics Committee of the First Affiliated Hospital of Zhengzhou University (2022-KY-0026-002).

### LIRI model

A mouse LIRI model was constructed as described in previous studies 23. Briefly, the mice were anesthetized via intraperitoneal injection of sodium pentobarbital (60 mg/kg) and restrained with the abdomen upright, and a median abdominal incision was made to fully expose the hepatic portal vessels. Ischemia was induced by occluding the blood supply to the left and middle lobes of the liver using a noninvasive vascular clamp. After 90 minutes of ischemia, the vascular clamp was removed and reperfusion was started. The mice in the sham-operated group did not undergo vascular occlusion and the remaining surgical procedures were the same as those used in the LIRI group. The mice were anesthetized and euthanised with sodium pentobarbital (200mg/kg) at different times after reperfusion, and serum and liver tissue samples were collected for further testing and analysis.

### Human liver samples

Human LIRI samples were collected from tissue biopsied 2-3 h after portal reperfusion from the livers of transplant recipients. Biopsies were collected from the left lobes of donor livers before orthotopic liver transplantation and used as control samples. All the subjects or their families signed informed consent forms. The studies were conducted in accordance with the principles of the Declaration of Helsinki. Permission to obtain and use human samples was obtained from the Ethics Committee of the First Affiliated Hospital of Zhengzhou University.

### Liver function analysis

Blood samples were collected and centrifuged at 3,000×g for 5 minutes, after which the supernatant was collected for analysis. Serum aspartate aminotransferase (AST) and alanine aminotransferase (ALT) levels were measured using kits according to the manufacturer's instructions (JianChen Bioengineering Institute, Nanjing, China).

### Hematoxylin and eosin staining

The liver tissues were fixed in 10% formalin for 48 hours, embedded in paraffin, cut into 5 μm thick sections. and then stained with hematoxylin and eosin (H&E) (Servicebio, Wuhan, China). Histopathological changes in the liver tissues were observed under a light microscope.

### Immunohistochemical staining

Paraffin-embedded sections were incubated at 60°C for 2 hours, dewaxed with xylene, dehydrated in gradient ethanol, subjected to ethylene diamine tetraacetic acid (EDTA) antigen retrieval and incubated with 3% hydrogen peroxide (H_2_O_2)_ at room temperature for 10 minutes to inactivate endogenous enzymes. The sections were blocked with 10% goat serum for 1 hour at room temperature, and incubated with primary antibody overnight at 4°C (Lg6g,1:200, Servicebio, Wuhan, China; SIRT4, 1:100, ABclonal, Wuhan, China). The following day, the sections were incubated with a secondary antibody for 30 minutes at room temperature. The sections were washed three times with phosphate-buffered saline (PBS) before and after incubation with secondary antibodies (Servicebio, Wuhan, China). Diaminobenzidine (DAB) chromogenic solution was added to the sections dropwise, followed by counterstaining with hematoxylin, sealing with neutral gum, and then observation under a light microscope.

### Quantitative real-time polymerase chain reaction (qRT-PCR)

TRIzol reagent (Solarbio, Beijing, China) was used to extract total RNA from liver tissue and cells, and a NanoDrop 2000 spectrophotometer was used to measure the amount of RNA extracted. Using a reverse transcription kit (Vazyme, Nanjing, China), 1 μg of total RNA was converted to complementary deoxyribonucleic acid (cDNA). cDNA was amplified on a qPCR apparatus using SYBR Green qPCR mix (Vazyme, Nanjing, China). Glyceraldehyde-3-phosphate dehydrogenase (GAPDH) served as the internal benchmark. The 2^-△△^CT approach was used for the analysis. The amplification primer sequences are provided in Supplementary Table 1.

### Western blot analysis

Proteins were extracted from cells and liver tissue using radio immunoprecipitation assay (RIPA) lysis buffer (Solarbio, Beijing, China), and protein concentrations were determined using bicinchoninic acid (BCA) assays (Solarbio, Beijing, China). Proteins were separated on sodium dodecylsulfate polyacrylamide gels and transferred to a polyvinylidene fluoride (PVDF) membrane. The membrane was incubated with 5% skim milk for 1 hour at room temperature and then incubated with primary antibody overnight at 4°C. Next, the membrane was incubated with secondary antibodies 1 hour at room temperature. An ImageQuant 800 system (Cytiva, America) was used to visualize and analyze the protein bands. Information on the antibodies is provided in Supplementary Table 2.

### Terminal deoxynucleotidyl transferase (TdT) dUTP nick-end labeling staining

Terminal deoxynucleotidyl transferase (TdT) dUTP Nick-End Labeling (TUNEL) Cell Apoptosis Detection Kit (Servicebio, Wuhan, China) was used for the experiment. The procedure was performed according to the manufacturer's protocols, and the images were observed and photographed under a fluorescence microscope.

### Single-cell RNA sequencing data processing

Single-cell RNA-sequencing (scRNA-seq) and data processing were performed and analyzed by Singleron (Nanjing, China). The detailed methods are shown in the Supporting Information.

### RNA-seq analysis

Total RNA was extracted from WT and SIRT4 KO mice subjected to LIRI, and cDNA libraries were constructed using a TruSeq Stranded mRNA LTSample Prep Kit (Illumina, San Diego, CA, USA) according to the manufacturer's instructions. Transcriptome sequencing and analysis were subsequently conducted by LC-Bio Co., Ltd (Hangzhou, China). Differential gene expression was analyzed using the DESeq package (2012) in R. The OmicStudio platform (https://www.omicstudio.cn/tool) was used for bioinformatic analysis.

### Measurement of oxidative stress indicators

Mouse liver tissues were homogenized at a ratio of weight (g): homogenate volume (mL) = 1:9, and the supernatant was collected after by centrifuging the homogenateation at 8000 round per minute for 10 min at 4 °C. The protein concentration was determined via a BCA assay kit. Malonaldehyde (MDA) and glutathione (GSH) levels, superoxide dismutase (SOD) and catalase (CAT) enzyme activities were measured in liver tissue using kits following according to the manufacturer's instructions (Beyotime, Shanghai, China).

### Analysis of ferrous iron content

Liver tissue was homogenized at a ratio of weight (g): homogenate volume (mL) = 1:9, and the supernatant was collected after centrifuging the homogenate at 12000×g for 10 minutes. The ferrous iron (Fe^2+^) levels in the liver tissue were determined according to the manufacturer's instructions (Elabscience, Wuhan, China), and the absorbance at 593 nm was measured on a microplate reader.

### Dihydroethidium staining

Optimal cutting temperature compound (OTC)-embedded liver tissues were cut into 5 μm-thick sections and stained with dihydroethidium (DHE) fluorescent probe (10 μM) for 30 minutes at room temperature, protected from light, and then stained nucleus with DAPI for 10 minutes, and the images were obtained under a fluorescence microscope.

### Transmission electron microscopy

Liver tissue (1 mm^3^ pieces) and AML12 cells were fixed in 4% glutaraldehyde at 4°C for 2 hours, washed with 0.1 mol/L phosphate buffer, and then fixed in 1% osmium tetroxide. Liver tissue and AML12 cells were dehydrated in an acetone gradient, permeabilized, embedded, and polymerized. The treated samples were cut into ultrathin sections approximately 70 nm thick, double stained with uranium lead, and observed under a transmission electron microscope (Hitachi, Tokyo, Japan).

### Cell culture and hypoxia-reoxygenation model

AML12 cells were obtained from Pricella Biotechnology Ltd. and cultured in Dulbecco's Modified Eagle Medium /F12 medium supplemented with 10% fetal bovine serum, 0.5% insulin-transferrin-selenium, 40 ng/mL dexamethasone, and 1% penicillin‒streptomycin (Procell, Wuhan, China). To establish the H/R model, the cell medium was replaced with glucose-free and serum-free DMEM/F12 (Procell, Wuhan, China). The cells were placed in a three-gas incubator containing 5% CO_2_, 94% N_2_, and 1% O_2_ at 37°C for 12 hours. At the end of the hypoxia period, the medium was replaced with complete medium and reoxygenated for different durations in a normoxic incubator.

### Plasmid and cell transfection

The plasmids used in this study were synthesized by OBiO Technology Co., Ltd. (Shanghai, China). The plasmids were transfected into HEK293T cells or AML12 hepatocytes with Lipo3000 transfection reagent according to the manufacturer's instructions (Thermo Scientific, USA). To establish stable SIRT4 overexpression and knockdown cell lines, SIRT4 lentiviral plasmids and lentiviral vectors were transfected into HEK293T cells to generate lentiviral particles, which were subsequently transfected into AML12 hepatocytes, and the cells were selected using medium containing 2 μg/mL puromycin for 7 days. The sequences of the lentiviral plasmids used for SIRT4 knockdown are listed in Supplementary Table 3.

### Detection of cell viability

Ninety-six-well plates (100 μL/well) were seeded with AML12 hepatocytes (0.5×10^4^ cells/mL). After reaching 70-80% confluence, and the cells were then exposed to a hypoxic environment. Cell counting kit-8 reagent (Solarbio, Beijing, China) was added to each well after reoxygenation, and the cells were incubated for 1 hour at 37°C. The absorbance at 450 nm was measured with a microplate reader.

### Immunoprecipitation (IP) and mass spectrometry assays

SIRT4-overexpressing AML12 cells were subjected to H/R, lysed with IP buffer for 30 min, and centrifuged at 12,000 × g for 10 min. Supernatants were acquired and incubated with protein A/G agarose beads and an anti-flag antibody overnight at 4°C. The beads were washed with IP buffer and boiled with 30 μL of protein loading buffer for 10 min at 100°C. Protein samples were used for Western blot and mass spectrometry analyses (completed by BMKcloud, Beijing, China).

### Molecular docking of SIRT4 and PRDX3

Protein-protein docking was performed to investigate the binding affinity between SIRT4 (AF-Q8R216-F1) and PRDX3 (AF-P20108-F1) by using the ClusPro2.0 server. ClusPro2.0 provides stiff docking directly using the protein-protein docking program (PIPER) tool, a docking software based on fast Fourier transform (FFT) algorithms that undertakes exhaustive sampling of the conformational space on a dense grid to sample the most near-native structures for a more accurate docking structure. The result was visualized using the molecular visualization tool PyMOL after the findings were obtained from the ClusPro service.

### Immunofluorescence (IF) staining

AML12 cells were fixed with 4% paraformaldehyde, permeabilized with 0.5% Triton for 5 minutes, blocked with 5% BSA for 30 minutes at room temperature, and incubated overnight at 4°C with anti-SIRT4 (1:200, ABclonal, Wuhan, China) and PRDX3 (1:200, Proteintech, Wuhan, China) primary antibody. After three washes with PBS, the cells were incubated with fluorescent secondary antibody for 1 hour at 37°C, and then the nuclei were stained with dihydrochloride (DAPI) for 10 minutes. Images were obtained under a fluorescence microscope. Paraffin-embedded sections were incubated at 60°C for 2 hours, dewaxed with xylene, dehydrated in gradient ethanol, and subjected to EDTA antigen retrieval. The sections were incubated with 10% goat serum for 1 hour at room temperature, followed by incubation with antibodies and DAPI and by imaging as for cells.

### Immunoprecipitation and coimmunoprecipitation (Co-IP)

Cells and liver tissues were lysed using IP lysis buffer (Beyotime, Shanghai, China) and centrifuged at 12,000 ×g at 4°C for 10 minutes to obtain protein lysates. The lysates were incubated overnight at 4°C with the corresponding primary antibody and protein A+G agarose beads (Beyotime, Shanghai, China). The beads were washed three times with IP lysis buffer and boiled with 2 × loading buffer at 100°C for 5 minutes to obtain the proteins. The proteins were subjected to immunoblot analysis.

### Liver-targeted LNPs sirt4 mRNA

Lipid nanoparticles (LNPs) were prepared by dissolving 1,2-dilinoleyloxy-3-dimethylaminopropane (Dlin-MC3-DMA), 1,2-distearoyl-sn-glycero-3-phosphocholine (DSPC), 1,2-Dimyristoylsnglycerol-3-methoxypolyethylene glycol (DMG-PEG), and cholesterol in ethanol, while messenger ribonucleic acid (mRNA) was dissolved in citrate buffer at a concentration of 0.1 mol/L with a pH of 4.5. The lipid and mRNA solutions were rapidly mixed in a 1:3 volume ratio. The initial formulation of the LNPs included SM102, DSPC, cholesterol, and DMG-PEG in a molar ratio of 50:10:38.5:1.5. The LNPs were then obtained by replacing the ethanol and citrate buffer with PBS through a process of ultrafiltration.

To evaluate the mRNA loading efficiency into LNPs, we prepared samples by mixing either 0.2 μg of free mRNA or LNPs-mRNA (containing 0.2 μg mRNA) with 2× RNA Loading Buffer (Denatured). Following a 10-minute incubation at 65°C, the samples were loaded onto a 1.0% denaturing formaldehyde agarose gel containing GelRed. mRNA sizes were determined using millennium™ RNA markers. The gels were then imaged using a ChemiDoc MP system from Bio-Rad in Hercules, CA, USA. Additionally, the encapsulation efficiency of the mRNA within the LNPs was quantified using a Quant-iT^TM^ RiboGreen™ RNA assay kit.

The size distribution and zeta potential of LNPs-mRNA were characterized using a Zetasizer Nano ZS90 (Malvern Instruments, located in Malvern, United Kingdom). These LNPs were stored at 4°C. Over a seven-day period, the storage stability of the LNPs-mRNA was assessed daily by monitoring changes in particle size and zeta potential. Each experiment was conducted in triplicate to ensure reliability.

Finally, the morphology of LNPs-mRNA was analyzed using transmission electron microscopy (Hitachi, Tokyo, Japan). For this, a drop of the LNPs-mRNA suspension was applied to copper electron microscopy grids, followed by negative staining with phosphotungstic acid to facilitate observation.

### Statistical analysis

All the data were statistically analyzed using SPSS (version 22.0; IBM, Armonk, NY, USA). GraphPad Prism 8.0 software was used for plotting graphs. The data are expressed as means ± standard deviations. Independent samples t tests were used for comparisons between two groups, and one-way ANOVA with Tukey's multiple comparison test for multiple group comparisons. Correlations were analyzed via Pearson's linear regression analysis. *P*<0.05 was considered statistically significant.

## Results

### Liver SIRT4 expression was downregulated after LIRI

**Figure 1:**
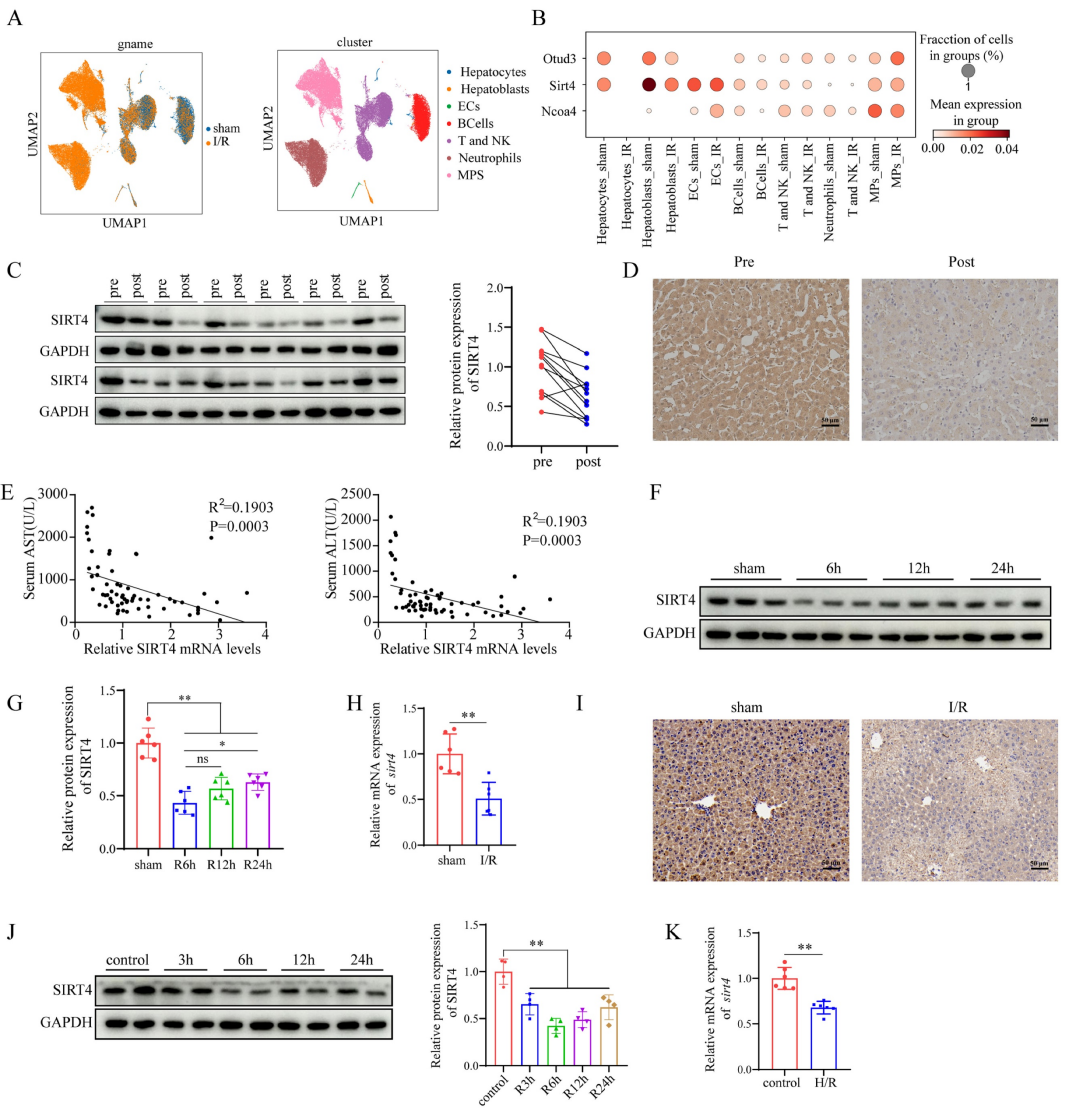
** LIRI decreases SIRT4 expression and enhances ferroptosis activation.** (A) UMAP of total cells from sham group and IR group. (B) Expression of the indicated gene in different cell subsets. (C) SIRT4 protein expression in the livers of human liver transplantation (LT) patients (n=12/group). (D) Immunohistochemical staining of SIRT4 in liver sections from human LT patients. (E) Correlation analysis of postoperative serum AST and ALT levels and liver SIRT4 mRNA levels (n=64). (F-G) SIRT4 protein expression in mouse liver tissue after different durations of reperfusion (n=6/group). (H) SIRT4 mRNA expression in mouse liver tissue after 6 h of reperfusion (n=6/group). (I) Immunohistochemical staining of SIRT4 in liver sections from mouse liver tissue after 6 h of reperfusion. (J) SIRT4 protein expression in AML12 cells after different durations of reoxygenation (n=4/group). (K) SIRT4 mRNA expression in mouse AML12 cells after 6 h of reoxygenation (n=6/group). The data are presented as mean ± SD. *P<0.05; **P<0.01.

Single-cell sequencing data revealed that SIRT4 expression was downregulated after LIRI (Figure 1A-B). The Western blot and IHC results revealed that SIRT4 protein levels were significantly lower in the posttransplantation samples than in the pretransplantation samples (Figure 1C-D). In addition, the correlation between the mRNA level of SIRT4 in liver tissue after liver transplantation and the postoperative liver function of patients was further analyzed and revealed a significant negative correlation with the serum ALT and AST levels (Figure 1E). Furthermore, Western blot and qPCR results revealed that the downregulation of SIRT4 expression decreased with increasing reperfusion time and peaked at 6 hours of reperfusion (Figure 1F-H). Furthermore, immunohistochemical staining of mouse liver sections revealed that SIRT4 expression was significantly lower in the I/R group than in the sham group (Figure 1I). Consistent with the *in vivo* results, SIRT4 expression was lower in AML12 hepatocytes challenged with H/R stimulation than in control cells, with the lowest expression at 6 hours after reperfusion (Figure 1J-K). In conclusion, these results suggest that SIRT4 may be involved in LIRI.

### SIRT4 deficiency aggravates LIRI *in vivo* and *in vitro*

**Figure 2:**
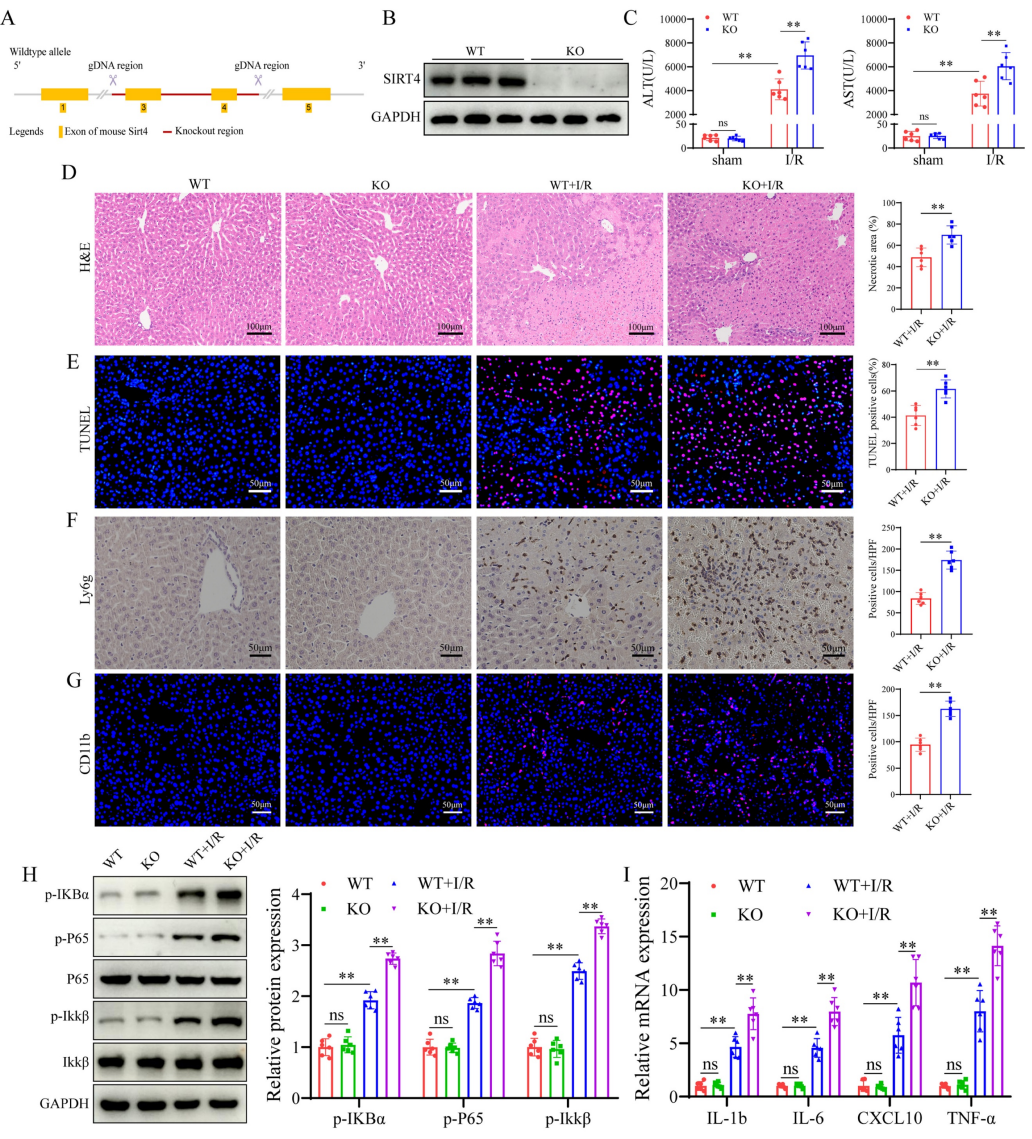
** SIRT4 deficiency aggravates liver I/R injury.** (A) Schematic diagram of the construction of SIRT4 KO mice. (B) SIRT4 protein expression in the liver tissues of WT and SIRT4 KO mice. (C) Serum ALT and AST levels in WT and SIRT4 KO mice subjected to different treatments (n=6/group). (D) H&E staining and necrotic area statistics of liver tissues in WT and KO mice subjected to sham or I/R treatment. (E) TUNEL staining and statistical analysis of liver tissue from WT and KO mice under different conditions (n=6/group). (F) Immunohistochemical staining and statistics of Ly6g positive inflammatory cells in liver tissue in WT and KO mice under different treatments (n=6/group). (G) Immunofluorescence staining and statistical analysis of CD11b positive inflammatory cells (red) in liver tissue from WT and KO mice (n=6/group). (H) NF-κB signaling protein detection and statistical analysis of liver tissues from WT and KO mice subjected to different treatments (n=6/group). (I) mRNA expression of the inflammatory cytokines IL-1β, IL-6, CXCL10 and TNF-α in the liver tissues of WT and SIRT4 KO mice subjected to different treatments (n=6/group). The data are presented as mean ± SD. *P<0.05; **P<0.01.

Following the observation of the downregulation of SIRT4 expression in LIRI, we investigated whether SIRT4 deficiency exacerbates LIRI. To explore this, SIRT4 KO mice were generated (Figure 2A-B), then SIRT4 KO and WT controls mice were subjected to LIRI. Compared with WT control mice, SIRT4 KO mice presented higher serum levels of ALT and AST (Figure 2C), accompanied by a marked increase in areas of hepatocyte necrosis (Figure 2D). TUNEL staining analysis revealed a greater incidence of hepatocellular death in the SIRT4 KO mice than in the WT mice following LIRI (Figure 2E). Immunohistochemistry and immunofluorescence revealed that the number of Ly6g and CD11b positive inflammatory cells was significantly increased in the SIRT4 KO mice than in the WT mice following LIRI (Figure 2F-G). Moreover, the activation of nuclear factor kappa-B (NF-κB) signaling was greater in the SIRT4 KO mice than in the WT mice after LIRI (Figure 2H). In addition, the mRNA expression levels of the proinflammatory cytokines IL-1β, IL-6, CXCL10 and TNF-α were substantially increased in the livers of the SIRT4 KO mice than in those of the WT mice (Figure 2I). Furthermore, *in vitro* cellular experiments also demonstrated the SIRT4 knockdown aggravated H/R-induced cell injury (Figure S1A-B). Together, these results show that SIRT4 deficiency accelerates LIRI.

### SIRT4 overexpression alleviates liver I/R injury *in vivo* and *in vitro*

**Figure 3:**
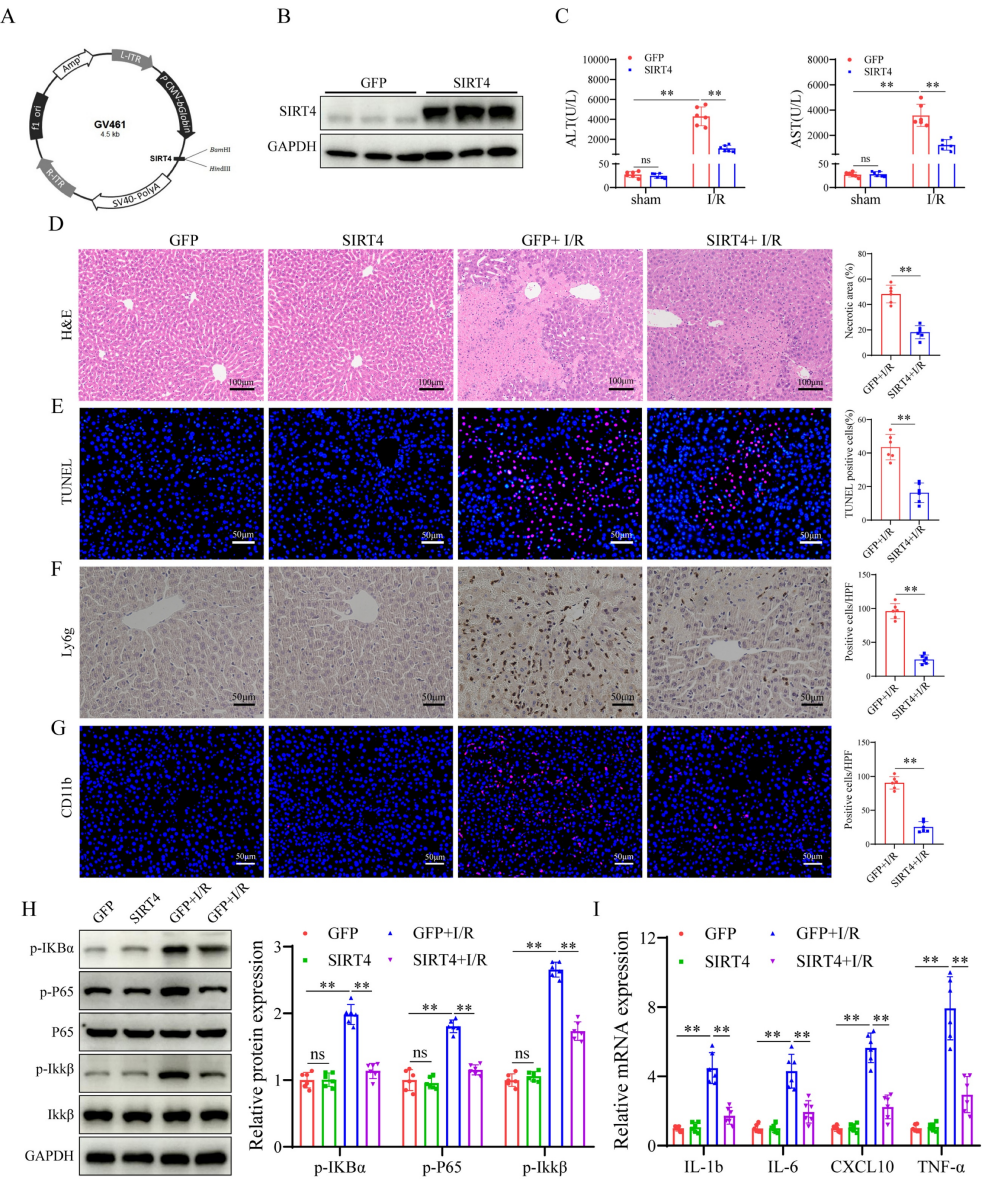
** SIRT4 overexpression alleviates liver I/R injury.** (A) Schematic diagram of the construction of AAV-SIRT4. (B) SIRT4 protein expression in the liver tissues of AAV-GFP and AAV-SIRT4 mice. (C) Serum ALT and AST levels in WT and SIRT4 KO mice subjected to different treatments (n=6/group). (D) H&E staining and necrotic area statistics of liver tissues from AAV-GFP and AAV-SIRT4 mice subjected to sham or I/R treatment (n=6/group). (E) TUNEL staining and statistical analysis of liver tissue from AAV-GFP and AAV-SIRT4 mice under different conditions (n=6/group). (F) Immunohistochemical staining and statistical analysis of Ly6g positive inflammatory cells in liver tissue from AAV-GFP and AAV-SIRT4 mice subjected to different treatments (n=6/group). (G) Immunofluorescence staining and statistical analysis of CD11b positive inflammatory cells (red) from AAV-GFP and AAV-SIRT4 mice subjected to different treatments (n=6/group). (H) NF-κB signaling protein detection and statistical analysis of liver tissues from WT and KO mice subjected to different treatments (n=6/group). (I) mRNA expression of the inflammatory cytokines IL-1β, IL-6, CXCL10 and TNF-α in liver tissues from AAV-GFP and AAV-SIRT4 treated mice (n=6/group). The data are presented as mean ± SD. *P<0.05; **P<0.01.

We further investigated the effect of SIRT4 on LIRI via the liver overexpression (OE) of SIRT4 in mice. Mice were injected with AAV8- SIRT4 (SIRT4-OE mice) or control AAV8-GFP via the tail vein (Figure 3A) and were challenged with I/R injury four weeks later. AAV8-SIRT4 markedly increased the liver protein levels of SIRT4 (Figure 3B). Compared with control mice, SIRT4-OE mice presented lower levels of ALT and AST after LIRI (Figure 3C). Histological assessment revealed less hepatocyte necrosis in the livers of the SIRT4-OE mice than in those of the GFP mice (Figure 3D). TUNEL staining analysis revealed a lower incidence of hepatocellular death in the SIRT4-OE mice than in the GFP mice following LIRI (Figure 3E). Immunohistochemistry and immunofluorescence revealed that the number of Ly6g and CD11b positive inflammatory cells was significantly lower in the SIRT4-OE mice than in the GFP mice following LIRI (Figure 3F-G). Additionally, the activation of NF-κB signaling was inhibited in the SIRT4-OE mice compared to WT mice after LIRI (Figure 3H). mRNA expression of genes associated with proinflammatory IL-1β, IL-6, CXCL10 and TNF-α was lower in the livers of SIRT4-OE mice than in those of GFP mice (Figure 3I). Furthermore, *in vitro* cellular experiments also demonstrated the protective role of SIRT4 against H/R-induced cell injury (Figure S1C-D). Together, these data indicate that the liver overexpression of SIRT4 ameliorates LIRI.

### SIRT4 negatively regulates ferroptosis during LIRI

**Figure 4:**
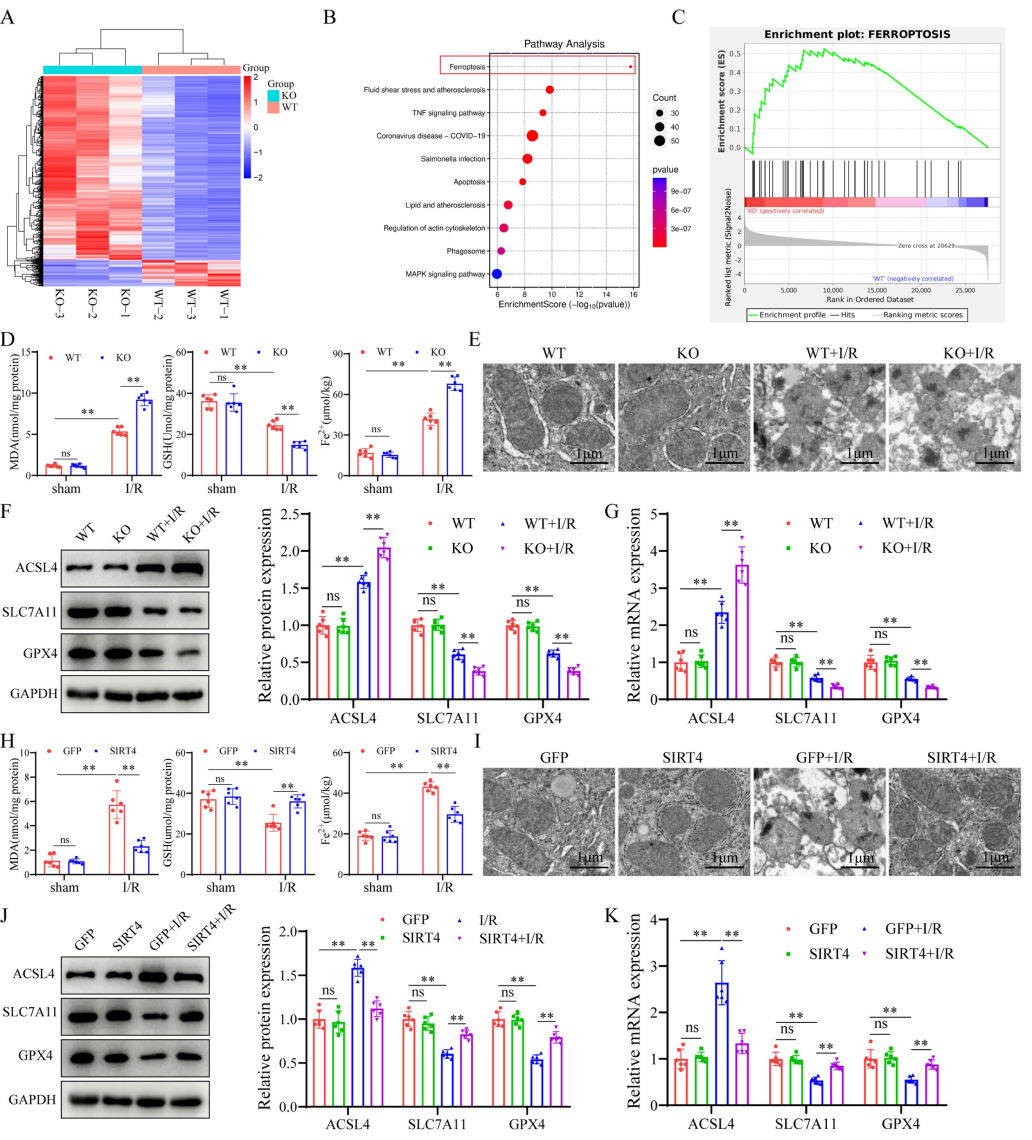
** SIRT4 negatively regulates ferroptosis during liver I/R injury. (A) Heatmap analysis of liver samples from WT and SIRT4 KO mice after LIRI. (B) KEGG enrichment scatter plot analysis of liver samples from WT and SIRT4 KO mice after LI**RI. (C) GSEA of ferroptosis signaling pathway components in WT and SIRT4 KO mice after LIRI. (D) MDA, GSH and Fe^2+^ contents in liver tissue from WT and SIRT4 KO mice subjected to different treatments (n=6/group). (E) Mitochondrial structure of WT and SIRT4 KO mice subjected to different treatments was observed via TEM (n=6/group). (F) Protein detection and statistical analysis of ACSL4, SLC7A11 and GPX4 in the liver tissues of WT and KO mice subjected to different treatments (n=6/group). (G) mRNA expression of ACSL4, SLC7A11 and GPX4 in the liver tissues of WT and KO mice subjected to different treatments (n=6/group). (H) MDA, GSH and Fe^2+^ contents in liver tissue from AAV-GFP and AAV-SIRT4 mice subjected to different treatments (n=6/group). (I) Mitochondrial structure of AAV-GFP and AAV-SIRT4 mice subjected to different treatments was observed via TEM. (J) Protein detection and statistical analysis of ACSL4, SLC7A11 and GPX4 in liver tissues from AAV-GFP and AAV-SIRT4 mice subjected to different treatments (n=6/group). (K) mRNA expression of ACSL4, SLC7A11 and GPX4 in liver tissue from AAV-GFP and AAV-SIRT4 mice subjected to different treatments (n=6/group). The data are presented as mean ± SD. *P<0.05; **P<0.01.

To determine the mechanism underlying the regulatory effect of SIRT4 on LIRI, we performed RNA sequencing using liver tissue from LIRI-challenged WT and SIRT4 KO mice. A heatmap defined the primary determinants of differences between liver samples from WT and SIRT4 KO mice (Figure 4A). Kyoto Encyclopedia of Genes and Genomes (KEGG) pathway enrichment analysis revealed that the ferroptosis signaling pathway was one of the most enriched pathways contributing to SIRT4-mediated LIRI (Figure 4B). Gene set enrichment analysis (GSEA) revealed that the ferroptosis pathway was activated in the SIRT4 KO mice during LIRI (Figure 4C). Therefore, we examined whether the ferroptosis pathway is required for the mediation of SIRT4 in response to LIRI. In this study, the levels or enzyme activities of GSH, SOD and CAT were lower, the MDA level, Fe^2+^ accumulation and DHE fluorescence intensity were significantly higher in the SIRT4 KO mice than in the WT mice following LIRI (Figure 4D, Figure S2A-B and S2E). Transmission electron microscopy revealed a greater degree of mitochondrial shrink and disruption of cristae in the SIRT4 KO group than in the WT group following LIRI (Figure 4E). Ferroptosis indicators revealed that the mRNA and protein levels of Acyl-CoA synthetase long-chain family member 4 (ACSL4) were significantly greater and that the protein and mRNA levels of solute carrier family 7 member 11 (SLC7A11) and glutathione peroxidase 4 (GPX4) were lower in the SIRT4 KO group than in the WT group following LIRI (Figure 4F-G). Conversely, compared with mice in the GFP group, mice in the liver-specific SIRT4 overexpression group had lower MDA, Fe^2+^ concentrations and DHE fluorescence intensity (Figure 4H, Figure S2F); higher GSH, SOD and CAT levels or enzyme activities (Figure 4H, Figure S2C-D); improved of mitochondrial morphology (Figure 4I); and reversed changes in the expression of ferroptosis indicator proteins and mRNA (Figure 4J-K). Further *in vitro* results indicated that SIRT4 knockdown in AML12 hepatocytes markedly increased the protein expression of ACSL4 and decreased the protein expression of SLC7A11 and GPX4 (Figure S2G). Additionally, SIRT4 knockdown markedly increased H/R-induced mitochondrial shrink and disruption of cristae in AML12 hepatocytes (Figure S2I); in contrast, SIRT4-overexpressing AML12 hepatocytes exhibited opposite changes after H/R (Figure S2H, S2J). Taken together, these results confirm that SIRT4 inhibits LIRI -induced ferroptosis.

### SIRT4 alleviates LIRI through suppression of ferroptosis

**Figure 5:**
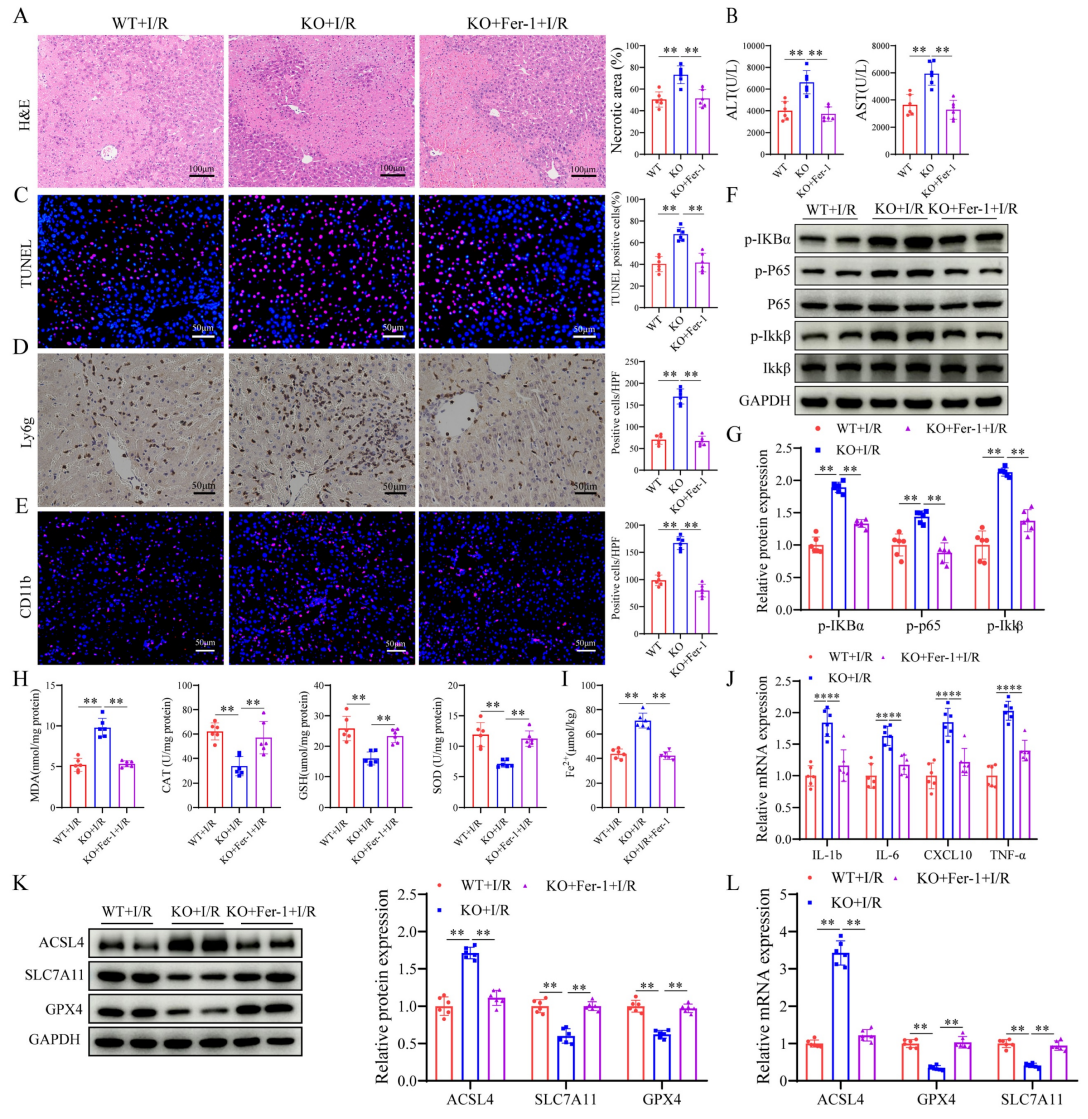
** SIRT4 deficiency induces liver I/R injury through ferroptosis.** (A) H&E staining and necrotic area statistics of liver tissue from WT and SIRT4 KO mice subjected to different treatments (n=6/group). (B) Serum ALT and AST levels in WT and SIRT4 KO mice subjected to different treatments (n=6/group). (C) TUNEL staining and statistical analysis of liver tissue from WT and SIRT4 KO mice subjected to different treatments (n=6/group). (D) Immunohistochemical staining and statistical analysis of Ly6g positive inflammatory cells in liver tissue from WT and KO mice subjected to different treatments (n=6/group). (E) Immunofluorescence staining and statistical analysis of CD11b positive inflammatory cells (red) in liver tissue from WT and KO mice subjected to different treatments (n=6/group). (F-G) NF-κB signaling protein detection and statistical analysis of liver tissue from WT and KO mice subjected to different treatments (n=6/group). (H) MDA, CAT, GSH and SOD levels or enzyme activities in liver tissue from WT and SIRT4 KO mice subjected to different treatments (n=6/group). (I) Liver Fe^2+^ content in WT and SIRT4 KO mice subjected to different treatments (n=6/group). (J) mRNA expression of the inflammatory cytokines IL-1β, IL-6, CXCL10 and TNF-α in liver tissue from WT and SIRT4 KO mice subjected to different treatments (n=6/group). (K) Protein detection and statistical analysis of ACSL4, SLC7A11 and GPX4 in liver tissue from WT and KO mice subjected to different treatments (n=6/group). (L) mRNA expression ACSL4, SLC7A11 and GPX4 in liver tissues from WT and KO mice subjected to different treatments (n=6/group). The data are presented as mean ± SD. *P<0.05; **P<0.01.

To investigate the role of ferroptosis in SIRT4-regulated LIRI, we pretreated SIRT4 KO mice with the ferroptosis inhibitor ferrostatin-1 (Fer-1, 10 mg/kg). We found that mice pretreated with Fer-1 had lower serum ALT and AST levels compared to SIRT4 KO mice subjected to LIRI (Figure 5B); furthermore, histological analysis revealed that the extent of hepatocyte necrosis was significantly improved (Figure 5A). TUNEL staining analysis revealed a lower incidence of hepatocellular death in Fer-1 treated mice than in SIRT4 KO mice following LIRI (Figure 5C). Immunohistochemistry and immunofluorescence revealed that the number of Ly6g and CD11b positive inflammatory cells was significantly lower in Fer-1 treated mice than in SIRT4 KO mice following LIRI (Figure 5D-E); additionally, the activation of NF-κB signaling was inhibited in Fer-1 treated mice (Figure 5F-G). The liver mRNA expression of genes associated with proinflammatory IL-1β, IL-6, CXCL10 and TNF-α was lower in the livers of Fer-1 treated mice than in those of SIRT4 KO mice (Figure 5J). The levels or enzyme activities of GSH, SOD and CAT were higher, MDA level, Fe^2+^ accumulation and DHE fluorescence intensity were significantly lower in Fer-1 treated mice than in the SIRT4 KO mice following LIRI (Figure 5H-I, Figure S3). Further, the expression levels of GPX4 and SLC7A11 decreased and the expression level ACSL4 increased after LIRI (Figure 5K-L). However, in Fer-1 treated mice, the expression of GPX4 and SLC7A11 was upregulated, and the expression of ACSL4 was downregulated (Figure 5K-L). Collectively, these data show that SIRT4 mitigates LIRI by suppressing ferroptosis in mice.

### SIRT4 deacetylates PRDX3 at K92 in LIRI

**Figure 6:**
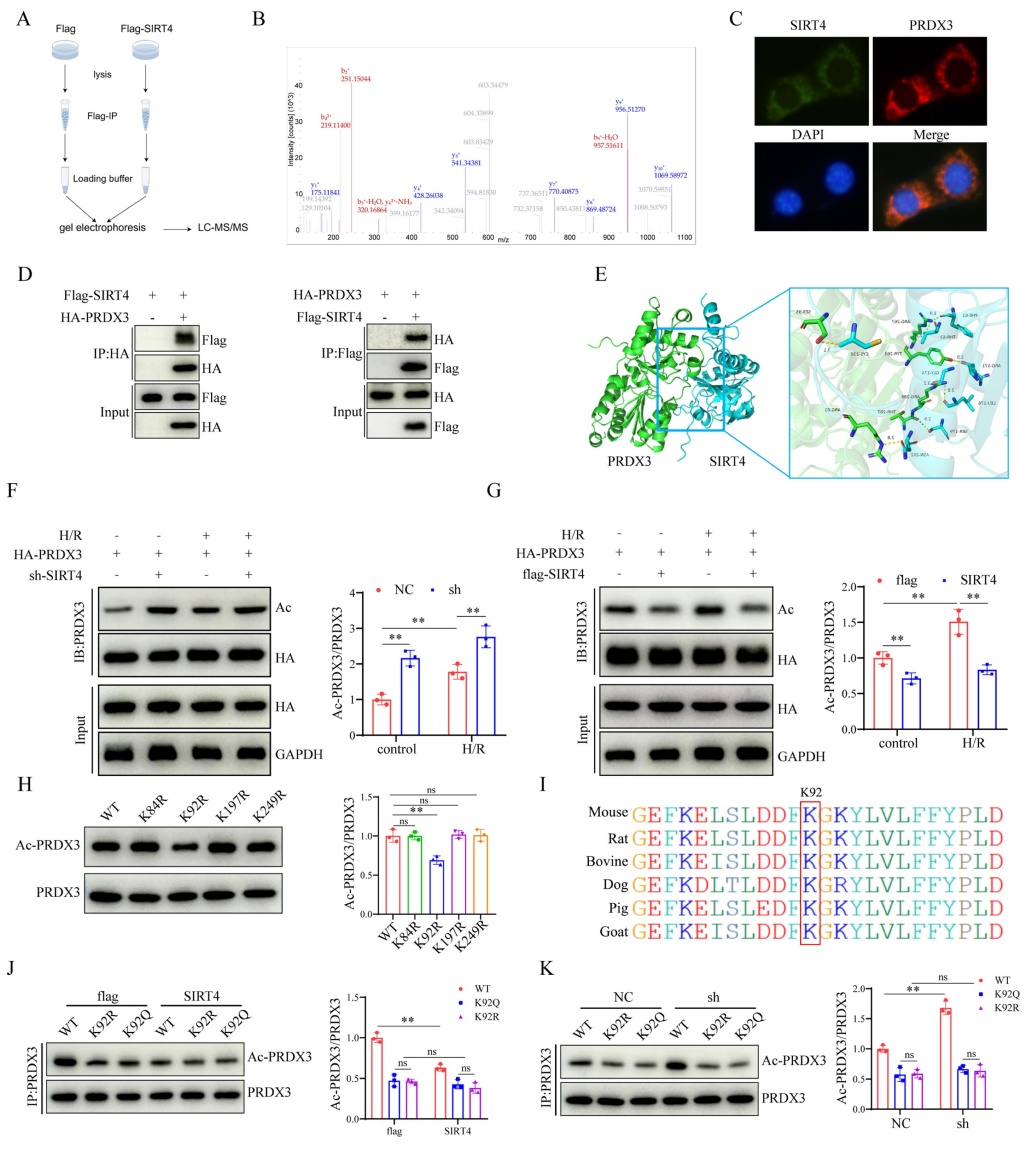
** SIRT4 deacetylates PRDX3 at K92 in LIRI.** (A) LC‒MS/MS analysis of the SIRT4-binding proteins from Flag and Flag-SIRT4 AML12 hepatocytes after H/R challenge. (B) LC‒MS/ MS spectrum of the PRDX3 protein. (C) Representative fluorescence images showing the colocalization of SIRT4 (green) and PRDX3 (red) in AML12 hepatocytes. (D) Flag-tagged SIRT4 and HA-tagged PRDX3 plasmids were cotransfected into HEK293T cells, Co-IP experiment analysis of the interaction between SIRT4 and PRDX3. (E) Molecular docking simulation of SIRT4 and PRDX3. (F) Expression and statistical analysis of acetylated PRDX3 protein in SIRT4 control and knockdown AML12 hepatocytes after H/R injury (n=3/group). (G) Expression and statistical analysis of acetylated PRDX3 protein in Flag and SIRT4 overexpressing AML12 hepatocytes after H/R injury (n=3/group). (H) Changes in the acetylation level, as determined by IP, in HEK293T cells transfected with HA-PRDX3, HA-PRDX3 (K84R), HA-PRDX3 (K92R), HA-PRDX3 (K197R), and HA-PRDX3 (K249R) plasmids (n=3/group). (I) The lysine K92 site of PRDX3 is highly conserved across different species. (J) Expression and statistical analysis of acetylated PRDX3 protein in Flag and SIRT4 overexpressing AML12 hepatocytes after transfection with HA-PRDX3 (WT), HA-PRDX3 (K92R), and HA-PRDX3 (K92Q) plasmids (n=3/group). (K) Expression and statistical analysis of acetylated PRDX3 protein in SIRT4 control and knockdown AML12 hepatocytes after transfection with HA-PRDX3 (WT), HA-PRDX3 (K92R), HA-PRDX3 (K92Q) plasmids (n=3/group). The data are presented as mean ± SD. *P<0.05; **P<0.01.

To explore the mechanism by which SIRT4 regulates ferroptosis, we performed a mass spectrometry assay to search for SIRT4-binding proteins (Figure 6A). The results identified PRDX3 as a candidate protein that interacts with SIRT4 (Figure 6B). We examined the localization of SIRT4 and PRDX3 and found that SIRT4 colocalized with PRDX3 (Figure 6C). Co-IP analysis confirmed the interaction between SIRT4 and PRDX3 (Figure 6D). Molecular docking experiments revealed a potential interaction between SIRT4 and PRDX3 (Figure 6E). Considering that SIRT4 is an important molecule that regulates acetylation, and that PRDX3 is also subject to acetylation regulation, changes in PRDX3 protein acetylation were detected. After cotransfection with PRDX3 and either knockdown or overexpression of SIRT4, SIRT4 knockdown significantly increased level of acetylated PRDX3 (Figure 6F); conversely, the level of acetylated PRDX3 further decreased after SIRT4 overexpression (Figure 6G). We subsequently investigated the SIRT4-targeted deacetylation sites in PRDX3. The PhosphoSitePlus database was used to analyze the lysine acetylation sites of PRDX3, and the results indicated that K84, K92, K197, and K249 were potential acetylation sites on PRDX3 in mice. Then, we transfected AML12 cells with individual PRDX3 lysine site-specific mutant plasmids (lysine (K) mutated to glutamine (Q) mimicking acetylation, and K mutated to arginine (R) mimicking deacetylation) and subjected the cells to H/R, and the results indicated that transfection with only the K92R (a highly conserved lysine residue among species) plasmid markedly reduced the acetylation level of PRDX3 (Figure 6H-I). In contrast, neither SIRT4 overexpression nor SIRT4 knockdown altered the acetylation level of PRDX3 in AML12 cells transfected with a K92Q plasmid or K92R plasmid (Figure 6J-K), which suggested that K92 might be the main target of SIRT4 on PRDX3 in AML12 cells. These results suggest that SIRT4 binds to PRDX3 and deacetylates it mainly at the K92 residue.

### Acetylation of PRDX3 at K92 negates its protective effect on LIRI

**Figure 7:**
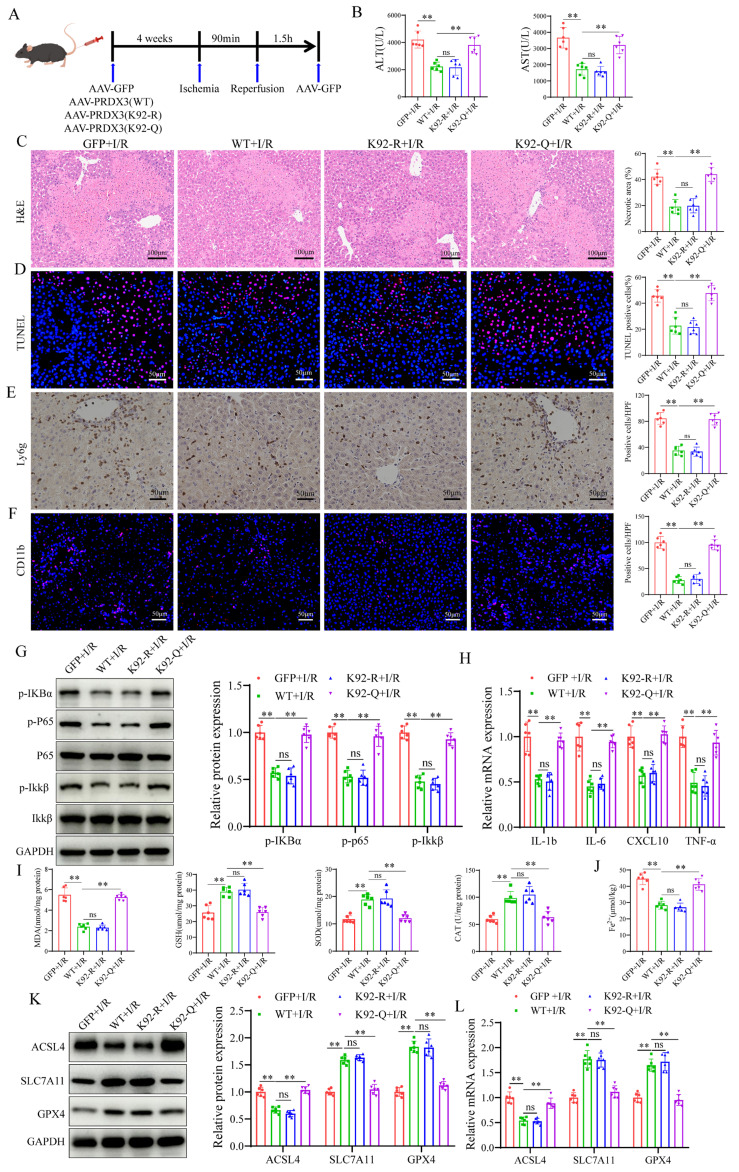
** Acetylation of PRDX3 at K92 negates its protective effect on LIRI.** (A) Schematic representation of adeno-associated virus intervention in mice and the I/R model. (B) Serum ALT and AST levels in different group of mice (n=6/group). (C) H&E staining and necrotic area statistics of liver tissue from different groups of mice (n=6/group). (D) TUNEL staining and statistical analysis of liver tissue from different groups of mice (n=6/group). (E) Immunohistochemical staining and statistical analysis of Ly6g positive inflammatory cells in liver tissue from different groups of mice (n=6/group). (F) Immunofluorescence staining and statistical analysis of CD11b positive inflammatory cells (red) in liver tissue from different groups of mice (n=6/group). (G) Detection and statistical analysis of NF-κB signaling proteins and statistical analysis in liver tissue from different groups of mice (n=6/group). (H) mRNA expression of the inflammatory cytokines IL-1β, IL-6, CXCL10 and TNF-α in liver tissues from the indicated groups (n=6/group). (I) MDA, GSH, SOD, CAT levels or enzyme activities in liver tissue from different groups of mice (n=6/group). (J) Liver Fe^2+^ levels in different groups of mice (n=6/group). (K) Protein detection and statistical analysis of ACSL4, SLC7A11 and GPX4 in liver tissues from different groups of mice (n=6/group). (L) mRNA expression of ACSL4, SLC7A11 and GPX4 in liver tissue from different groups of mice (n=6/group). The data are presented as mean ± SD. *P<0.05; **P<0.01.

To examine the role of PRDX3-K92 deacetylation in LIRI, AAV-GFP, AAV- PRDX3-WT, AAV-PRDX3-K92-R, and AAV-PRDX3-K92-Q overexpressed transfected mice were generated and then subjected to I/R (Figure 7A), and western blot results confirmed overexpression of these viruses in the liver (Figure S4A). The data indicated that transaminase (ALT/AST) levels (Figure 7B), liver necrotic areas (Figure 7C), hepatocellular death (Figure 7D), the number of inflammatory cell infiltration and NF-kB signaling pathway activation were greater in the GFP and PRDX3-92K-Q mice than in the PRDX3-WT and PRDX3-K92-R mice after LIRI (Figure 7E-H). Moreover, after LIRI, compared with that in PRDX3-WT and PRDX3-K92-R mice, I/R-induced ferroptosis were aggravated in GFP and PRDX3-K92-Q mice (Figure 7I-L, Figure S4B). Collectively, these data suggest that the deacetylation of PRDX3 at the K92 residue protects mice from LIRI.

### The protective effect of SIRT4 against LIRI depends on the deacetylation of PRDX3 at K92

**Figure 8:**
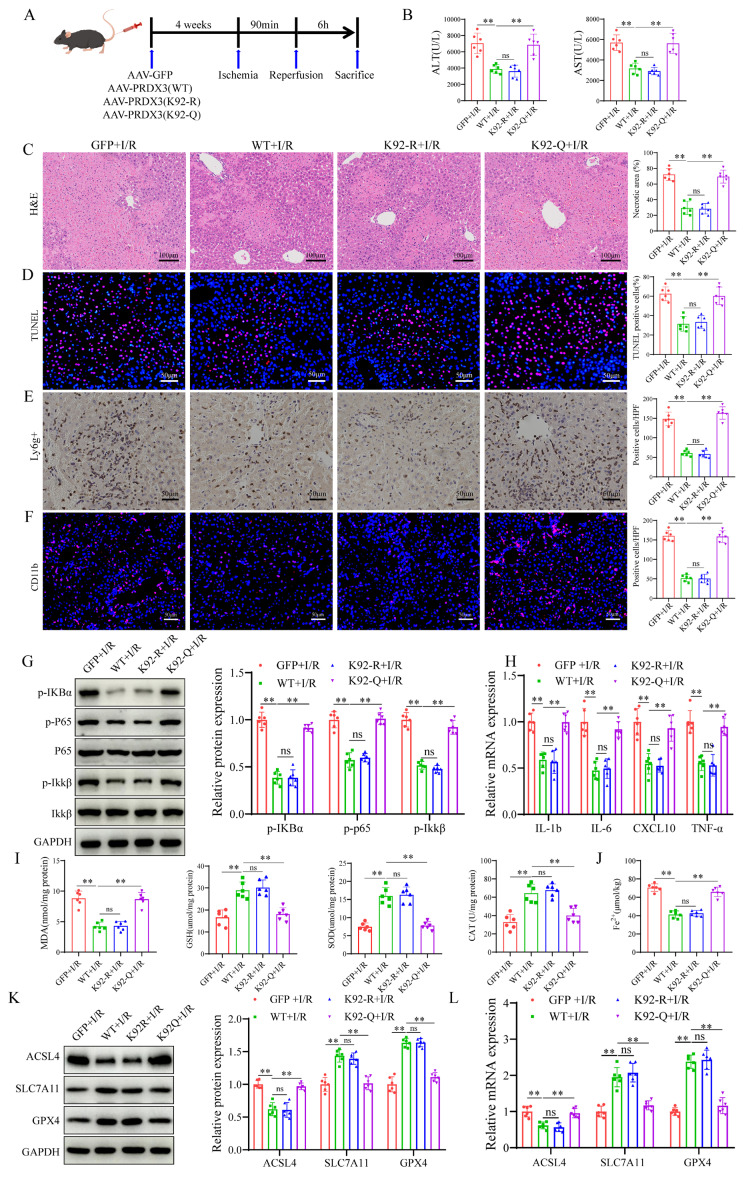
** The protective effect of SIRT4 against LIRI depends on the deacetylation of PRDX3 at K92.** (A) Schematic representation of adeno-associated virus intervention in SIRT4 KO mice and the I/R model. (B) Serum ALT and AST levels in SIRT4 KO mice subjected to different treatments (n=6/group). (C) H&E staining and necrotic area statistics of liver tissue from SIRT4 KO mice subjected to different treatments (n=6/group). (D) TUNEL staining and statistical analysis of liver tissue from SIRT4 KO mice subjected to different treatments (n=6/group). (E) Immunohistochemical staining and statistical analysis of Ly6g positive inflammatory cells in liver tissue from SIRT4 KO mice subjected to different treatments (n=6/group). (F) Immunofluorescence staining and statistical analysis of CD11b positive inflammatory cells (red) in liver tissue from SIRT4 KO mice subjected to different treatments (n=6/group). (G) Detection of NF-κB signaling proteins and statistical analysis of liver tissue from SIRT4 KO mice subjected to different treatments (n=6/group). (H) mRNA expression of the inflammatory cytokines IL-1β, IL-6, CXCL10 and TNF-α in the liver tissue of SIRT4 KO mice subjected to different treatments (n=6/group). (I) MDA, GSH, SOD, CAT levels or enzyme activities in liver tissue from SIRT4 KO mice subjected to different treatments. (J) Liver Fe^2+^ levels in SIRT4 KO mice subjected to different treatments (n=6/group). (K) Protein detection and statistical analysis of ACSL4, SLC7A11 and GPX4 in liver tissue from SIRT4 KO mice subjected to different treatments (n=6/group). (L) mRNA expression of ACSL4, SLC7A11 and GPX4 in liver tissue from SIRT4 KO mice subjected to different treatments (n=6/group). The data are presented as mean ± SD. *P<0.05; **P<0.01.

To investigate the role of PRDX3 deacetylation by SIRT4 in LIRI, we overexpressed various forms of PRDX3 in SIRT4 KO mice by injecting relevant AAVs expressing AAV-GFP, AAV-PRDX3-WT, AAV- PRDX3-K92-R, or AAV- PRDX3-K92-Q via the tail vein (Figure 8A), and western blot results confirmed overexpression of these viruses in the liver (Figure S5A); these mice were subsequently subjected to I/R. SIRT4 deficiency robustly increased serum transaminase (ALT/AST) levels and induced necrosis and hepatocellular death after LIRI, and PRDX3-WT overexpression significantly ameliorated liver damage (Figure 8B-C). In I/R-treated SIRT4 KO mice, neither the AAV-GFP nor the AAV-PRDX3-K92-Q group exhibited obvious improvements in serum transaminase (ALT/AST) levels, liver necrotic areas and hepatocellular death compared with those in the AAV-PRDX3-WT and AAV-PRDX3-K92-R group (Figure 8B-D). In contrast to those in the AAV-GFP mice, liver inflammation and ferroptosis were markedly inhibited in the AAV-PRDX3-WT and AAV-PRDX3-K92-R mice, but not in the GFP and AAV-PRDX3-K92-Q mice (Figure 8E-L, Figure S5B). Collectively, these data suggest that the deacetylation of PRDX3 at K92 mediates the protective effect of SIRT4 against LIRI.

### Liver-targeted lipid nanoparticles-sirt4 mRNA alleviates liver I/R injury

**Figure 9:**
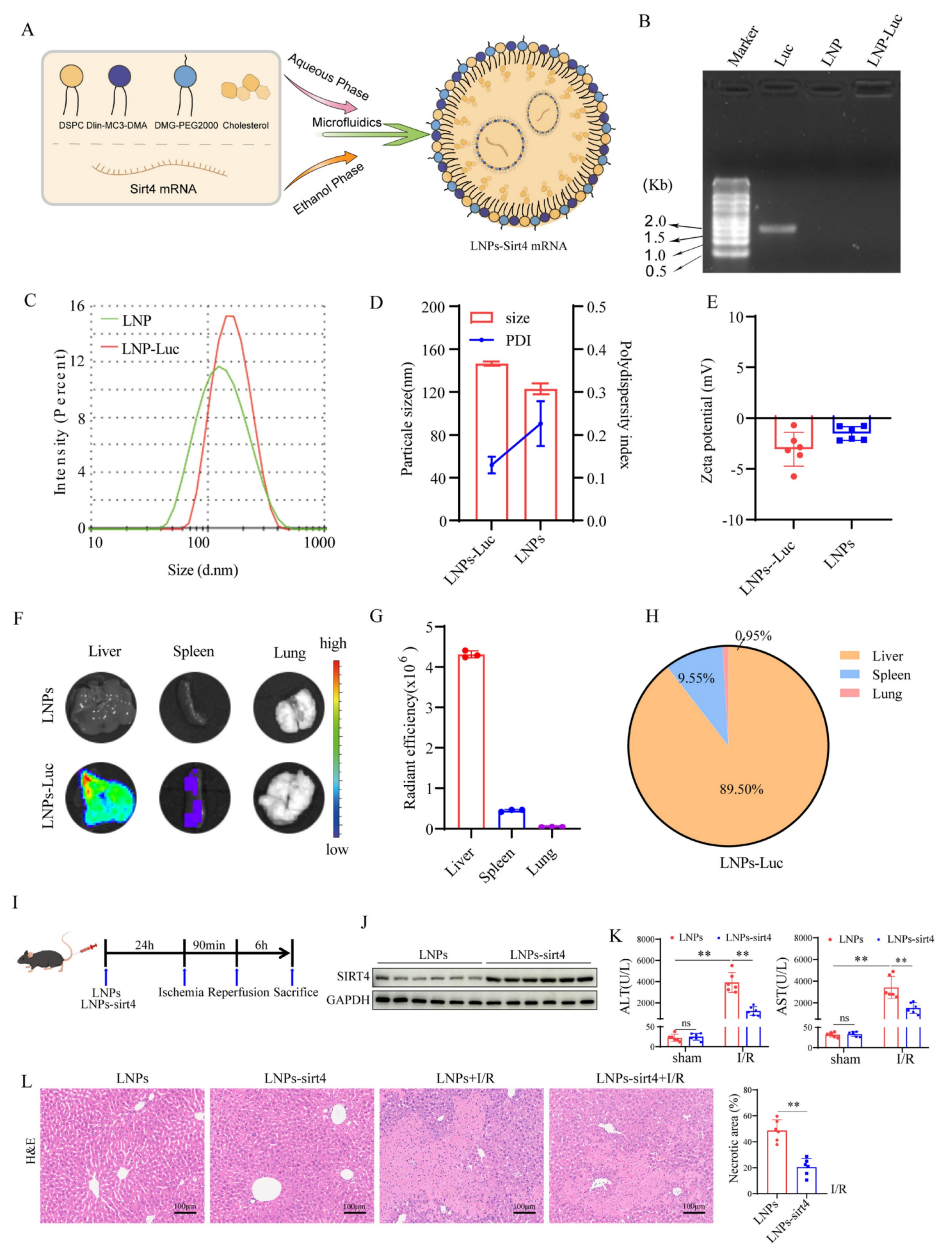
** Liver-targeted LNPs-sirt4 mRNA alleviates liver I/R injury.** (A) Schematic representation of the mRNA-loaded LNPs preparation. (B) The loading capacity of LNPs for Luc-mRNA was determined via agarose gel electrophoresis. (C and D) The particle size, (D) polydispersity index and (E) zeta potential of LNPs-based gene delivery systems was determined via dynamic light scattering. (F) Fluorescence imaging of organs isolated from C57BL/6 mice 24 h after the intravenous injection of LNPs loaded with Luc-mRNA. (G) Quantification of the fluorescence expression in the liver, spleen, and lung. (H) Percentage of fluorescence expression in the liver, spleen, and lung (n=3 independent samples). (I) Schematic representation of LNPs-sirt4 intervention in mice and I/R model. (J) Western blot analysis of SIRT4 protein expression in the livers of mice treated with LNP-sirt4 mRNA at 24 h postinjection (n=6/group). (K) Serum ALT and AST levels in different groups of mice (n=6/group). (L) H&E staining and necrotic area statistics of liver tissue from different mice in the indicated groups (n=6/group). The data are presented as mean ± SD. *P<0.05; **P<0.01.

To explore the therapeutic effects of a SIRT4 activator on LIRI, we developed liver-targeted lipid nanoparticles (LNPs)-sirt4 mRNA (Figure 9A). Agarose gel electrophoresis revealed that LNPs had good loading capacity for Luc-mRNA (Figure 9B). The particle size, polydispersity index (PDI) and zeta potential of LNP-sirt4 mRNA were measured via dynamic light scattering (Figure 9C-E). Mouse *in vivo* imaging revealed that LNPs-sirt4 mRNA mostly aggregated in the liver, indicating good liver targeting ability (Figure 9F-H). We then assessed the effect of LNPs-sirt4 mRNA on LIRI (Figure 9I). The protein expression level of SIRT4 was elevated in the livers of mice treated with LNPs-sirt4 mRNA (Figure 9J). Intriguingly, compared with those in the LNPs group, the serum AST, ALT levels and liver necrotic areas in the LNPs-sirt4 mRNA group were lower after LIRI challenge (Figure 9K-L). TUNEL staining revealed a reduction in hepatocellular death in liver sections from mice treated with LNPs-sirt4 mRNA (Figure S6A). Immunohistochemistry and immunofluorescence staining, protein expression of NF-κB signaling and mRNA expression of genes associated with proinflammatory also revealed that LNPs-sirt4 mRNA inhibited I/R-induced inflammation (Figure S6B-E). The levels or enzyme activities of GSH, SOD and CAT were higher, MDA level, Fe^2+^ accumulation and DHE fluorescence intensity were significantly lower in the LNPs-sirt4 mRNA group than in the LNPs group following LIRI (Figure S6F-K). Moreover, Western blot and qPCR analysis revealed an increase in the levels of the ferroptosis antagonism markers GPX4 and SLC7A11, and a decrease in ACSL4 and acetylated PRDX3 in LNPs-sirt4 mRNA group than in the LNPs group following LIRI, indicating that LNPs-sirt4 mRNA inhibited LIRI-induced ferroptosis and PRDX3 protein acetylation (Figure S6L-N). In summary, these findings strongly indicate that the overexpression of SIRT4 by LNPs-sirt4 mRNA attenuates LIRI, suggesting that liver-targeted LNPs-sirt4 mRNA is a promising means for the clinical treatment of LIRI.

## Discussion

LIRI is a complex pathophysiological process that strongly affects the prognosis of patients after liver transplantation and other liver surgeries. Despite extensive research into the mechanisms and treatment strategies for LIRI, there is still a lack of effective treatments in clinical practice. The present study provides evidence that SIRT4 expression is decreased in liver transplant patients, mice with I/R injury and AML12 hepatocyte challenged with H/R. Furthermore, SIRT4 KO dramatically exacerbated liver injury and ferroptosis in mice after LIRI, whereas SIRT4 overexpression produced the opposite results. Further investigation revealed that PRDX3 was a downstream target of SIRT4. Moreover, the K92 lysine residue in PRDX3 was deacetylated by SIRT4, resulting in the suppression of ferroptosis. Additionally, we found that liver-targeted LNPs-sirt4 mRNA effectively attenuated LIRI. These results suggest that SIRT4 may be an important target for alleviating LIRI.

SIRT4 is an nicotinamide adenine dinucleotide (NAD)-dependent deacetylase that is predominantly found in the mitochondria and plays diverse roles through posttranslational modifications in both physiological and pathological processes 24, 25. The deacetylase activity of SIRT4 has significant implications for the structure and function of various proteins, playing crucial roles in metabolic regulation by modulating fatty acid oxidation, insulin secretion, and energy homeostasis 20, 21, 25. Notably, SIRT4 is involved in the regulation of liver disease. For example, glutamine promotes the function of SIRT4 and modulates heat shock protein 60 (HSP60) deacetylation to protect hepatocytes from burn sepsis injury 26. Although SIRT4 is involved in the pathophysiological processes of liver diseases, its role in LIRI remains unknown. Our study is the first to explore the role of SIRT4 in LIRI, and the results demonstrated that SIRT4 exerts hepatoprotective effects by regulating protein deacetylation.

Ferroptosis, a form of programmed cell death characterized by iron accumulation and the depletion of plasma membrane polyunsaturated fatty acids, has been linked to various pathological conditions including neurological disorders, metabolic disturbances, I/R injury, and inflammation 27-29. As a new mode of cell death, the role of ferroptosis in disease has attracted considerable attention 30, 31. Emerging research has shown that SIRT4 is involved in the regulation of ferroptosis. Liu *et al.* showed that SIRT4 attenuates acute pancreatitis injury by inhibiting ferroptosis through the regulation of the hypoxia inducible factor-1α/ Heme Oxygenase-1 (HIF-1α/HO-1) pathway 19. Li *et al.* demonstrated that SIRT4 regulates glyceronephosphate O-acyltransferase acetylation and protein levels to attenuate ferroptosis in a mouse model of chronic obstructive pulmonary disease (COPD) induced by cigarette smoke particulates 32. Consistent with the results of those, our research demonstrated that SIRT4 KO mice exhibited increased levels of ferroptosis following LIRI. Conversely, AAV-mediated SIRT4 overexpression showed a reduction in ferroptosis after LIRI. In addition, ferrostatin-1 inhibited SIRT4 KO resulted in exacerbation of liver injury and ferroptosis. These findings were further supported by *in vitro* experiments, confirming the protective role of SIRT4 in mitigating LIRI-induced ferroptosis in mice.

Acetylation is a posttranslational modification in which acetyl groups are transferred and added to protein lysine residues under the catalysis of acetyl transferase 33. Acetylation can affect protein structure and function, and then regulate cell signaling, gene expression, protein stability and other biological processes 34, 35. Acetylation modification has been implicated in a wide range of liver diseases. In alcoholic liver disease, increasing the deacetylase activity of SIRT6 reduces the level of acetylated nuclear factor erythroid 2-related factor 2 (NRF2), thereby increasing the stability of NRF2, and thus attenuating hepatocyte damage and oxidative stress 36. The inhibition of sirtuin 2 (SIRT2) induces the acetylation and proteasomal degradation of fibrinogen-like protein 1, thus promoting cancer immunotherapy 37. Protein acetylation is also involved in LIRI. SIRT3 inhibits HIF-1α transcription, ROS levels, and the inflammatory response in macrophages by decreasing the acetylation, ubiquitination and degradation of forkhead box O3 (FOXO3) to alleviate LIRI 38. These findings suggest that protein acetylation may play a critical role in the pathogenesis of LIRI and that targeted protein acetylation may serve as a novel therapeutic strategy to alleviate LIRI.

Peroxiredoxins (Prxs) are conserved and ubiquitous thiol peroxidases that provide a primary defense against peroxides 39. Mammals have six Prx members (PRDX1-6), and PRDX3 was recently reported to suppress mitochondrial ROS by eliminating approximately 90% of hydrogen peroxide (H_2_O_2_) in the mitochondrial matrix, which suggests that it is a potent antioxidant enzyme that protects against mitochondrial oxidative damage 40, 41. In addition, PRDX3 can regulate the ferroptosis process in several diseases, and is a specific marker of ferroptosis 40, 42, 43. Moreover, the posttranslational modification of the PRDX3 protein, including acetylation, succinylation, and ubiquitination, is closely related to its function 44-46. Studies have shown that the SIRT3-mediated deacetylation of PRDX3 can reduce mitochondrial oxidative damage and apoptosis induced by intestinal I/R 45. Through immunoprecipitation and mass spectrometry assays, we identified PRDX3 as a candidate protein that interacts with SIRT4. Co-IP experiments and molecular docking demonstrated an interaction between SIRT4 and PRDX3. Given the deacetylation activity of SIRT4, whether PRDX3 also undergoes acetylation in LIRI, thus affecting its function has not been investigated. In this study, we found that PRDX3 interacted with SIRT4 and that SIRT4 KO increased the I/R-induced PRDX3 protein acetylation. Conversely, SIRT4 overexpression decreased PRDX3 protein acetylation. The PhosphoSitePlus database analysis revealed that lysine residues K84, K92, K197, and K249 are potential acetylation sites on PRDX3 in mice 47. We constructed mutation plasmids for potential PRDX3 acetylation sites and confirmed through IP experiments that SIRT4 deacetylates PRDX3 at the K92 site. To further validate that finding, we constructed AAV-PRDX3 (K92-R) and AAV-PRDX3 (K92-Q) viruses, conducted *in vivo* experiments in WT and SIRT4 KO mice, and confirmed that AAV-PRDX3 (K92-R) exerts therapeutic effects, manifesting as reduced liver injury and ferroptosis. Therefore, the K92 residue may be the dominant functional acetylation site on PRDX3 that is regulated by SIRT4 in LIRI.

SIRT4 depletion exacerbates LIRI and is accompanied by a significant increase in protein acetylation. In contrast, SIRT4 overexpression attenuates liver injury and reduces protein acetylation levels. Therefore, reversing the I/R-induced reduction in SIRT4 expression may be a promising strategy for ameliorating LIRI. We constructed liver-target NLPs-sirt4 mRNA to increase sirt4 expression in the liver. By using NLPs-sirt4 mRNA, we successfully restored SIRT4 expression levels in mice, decreasing ferroptosis, and thereby alleviating LIRI, indicating that exogenous SIRT4 overexpression is a promising strategy for the clinical treatment of LIRI.

However, this study still has limitations. The results would be more convincing if the study had used SIRT4 liver-specific KO mice and transgenic mice. Furthermore, the interaction domains between SIRT4 and PRDX3 have not been fully determined.

## Conclusions

In summary, our data demonstrate that SIRT4 alleviates LIRI by inhibiting ferroptosis in the liver. PRDX3 is a downstream target of SIRT4, and K92 in PRDX3 is deacetylated by SIRT4, thus resulting in the inhibition of ferroptosis. These findings strongly suggest that SIRT4 may be a drug target for developing agents to treat LIRI.

## Supplementary Material

Supplementary methods, figures and tables.

## Abbreviations

SIRT4: sirtuins
PRDX3: peroxiredoxins 3
LIRI: liver ischemia-reperfusion injury
AML12: alpha mouse liver 12
H/R: hypoxia-reoxygenation
WT: wild type
KO: knockout
Ac: acetylation
GFP: green fluorescent protein
MDA: malonaldehyde
GSH: glutathione
SOD: superoxide dismutase
CAT: catalase
ALT: alanine aminotransferase
AST: aspartate aminotransferase
H&E: hematoxylin and eosin
IHC: immunohistochemical staining
qRT-PCR: quantitative real-time polymerase chain reaction
TUNEL: terminal deoxynucleotidyl transferase (TdT) dUTP Nick-End Labeling
Fe^2+^: ferrous iron
Co-IP: immunoprecipitation and coimmunoprecipitation
GSEA: Gene set enrichment analysis
KEGG: Kyoto Encyclopedia of Genes and Genomes
TEM: Transmission electron microscopy
LNPs: Lipid nanoparticles
ACSL4: Acyl-CoA synthetase long-chain family member 4
GPX4: glutathione peroxidase 4
SLC7A11: solute carrier family 7 membrane 11
NF-Κb: nuclear factor kappa-B
GAPDH: Glyceraldehyde-3-phosphate dehydrogenase
K: lysine
R: arginine
Q: glutamine
