# Supplementary material for: SIRT4-Mediated Deacetylation of PRDX3 Attenuates Liver Ischemia Reperfusion Injury by Suppressing Ferroptosis
Source: Int J Biol Sci. 2025 Jul 11;21(10):4663–82. doi: 10.7150/ijbs.114510 (PMC12320503; doi:10.7150/ijbs.114510)
Supplement: Supplementary file 1 — Supplementary methods, figures and tables. [file ijbsv21p4663s1.pdf]

SIRT4-mediated deacetylation of PRDX3 attenuates liver ischemia reperfusion injury by suppressing ferroptosis

Materials and Methods

10×Genomics Chromium single-cell RNA sequencing data processing

Tissue dissociation and preparation

After reperfusion liver tissue obtained and were stored in the sCelLive™ Tissue Preservation Solution (Singleron) on ice after the surgery within 30 mins. The specimens were washed with Hanks Balanced Salt Solution (HBSS) for three times, minced into small pieces, and then digested with 3 mL sCelLive™ Tissue Dissociation Solution (Singleron) by Singleron PythoN™ Tissue Dissociation System at 37 °C for 15 min. The cell suspension was collected and filtered through a 40-micron sterile strainer. Afterwards, the GEXSCOPE® red blood cell lysis buffer (RCLB, Singleron) was added, and the mixture [Cell: RCLB=1:2 (volume ratio)] was incubated at room temperature for 5-8 min to remove red blood cells. The mixture was then centrifuged at 300×g 4 °C for 5 mins to remove supernatant and suspended softly with PBS. Finally, the samples were stained with Trypan Blue and the cell viability was evaluated microscopically.

RT & Amplification & Library Construction

Single-cell suspensions (2×10^5 cells/mL) with PBS were loaded onto microwell chip using the Singleron Matrix® Single Cell Processing System. Barcoding Beads are subsequently collected from the microwell chip, followed by reverse transcription of the mRNA captured by the Barcoding Beads and to obtain cDNA, and PCR amplification. The amplified cDNA is then fragmented and ligated with sequencing adapters. The scRNA-seq libraries were constructed according to the protocol of the GEXSCOPE® Single Cell RNA Library Kits (Singleron). Individual libraries were

diluted to 4 nM, pooled, and sequenced on Illumina novaseq 6000 with 150 bp paired end reads.

Primary analysis of raw read data

Raw reads from scRNA-seq were processed to generate gene expression matrixes using CeleScope (https://github.com/singleron-RD/CeleScope) v1.9.0 pipeline. Briefly, raw reads were first processed with CeleScope to remove low quality reads with Cutadapt v1.17 to trim poly-A tail and adapter sequences. Cell barcode and UMI were extracted. After that, we used STAR v2.6.1a to map reads to the reference genome GRCm38 (ensembl version 92 annotation). UMI counts and gene counts of each cell were acquired with featureCounts v2.0.1 software, and used to generate expression matrix files for subsequent analysis.

Quality control, dimension-reduction and clustering

Cells were filtered by gene counts below 200 and the top 2% gene counts and the top 2% UMI counts. Cells with over 20% mitochondrial content were removed. We used functions from Seurat v3.1.2 for dimension-reduction and clustering. Then we used NormalizeData and ScaleData functions to normalize and scale all gene expression, and selected the top 2000 variable genes with FindVariableFeautres function for PCA analysis. Using the top 20 principle components, we separated cells into multiple clusters with FindClusters. Batch effect between samples was removed by Harmony. Finally, UMAP algorithm was applied to visualize cells in a two-dimensional space.

Differentially expressed genes (DEGs) analysis

To identify differentially expressed genes (DEGs), we used the Seurat FindMarkers function based on Wilcox likelihood-ratio test with default parameters, and selected the genes expressed in more than 10% of the cells in a cluster and with an average log (Fold Change) value greater than 0.25 as DEGs. For the cell type annotation of each cluster, we combined the expression of canonical markers found in the DEGs with knowledge

from literatures, and displayed the expression of markers of each cell type with heatmaps/dot plots/violin plots that were generated with Seurat DotPlot/Vlnplot function. Doublet cells were identified as expressing markers for different cell types, and removed manually.

Cell type annotation

The cell type identity of each cluster was determined with the expression of canonical markers found in the DEGs using SynEcoSys database. Dot plots and violin plots displaying the expression of markers used to identify each cell type were generated by Seurat v3.1.2 DotPlot/Vlnplot.

Statistics and repeatability

Cell distribution comparisons between two groups were performed using unpaired two-tailed Wilcoxon rank-sum tests. Comparisons of gene expression or gene signature between two groups of cells were performed using unpaired two-tailed Student's t test. Comparisons of cell distribution of paired group1 and group2 as well as sham and I/R were performed using paired two-tailed Wilcoxon rank-sum tests. All statistical analyses and presentation were performed using R. Statistical tests used in figures were shown in figure legends and statistical significance was set at $P < 0.05$.

Supplementary figures

Supplementary Figure 1

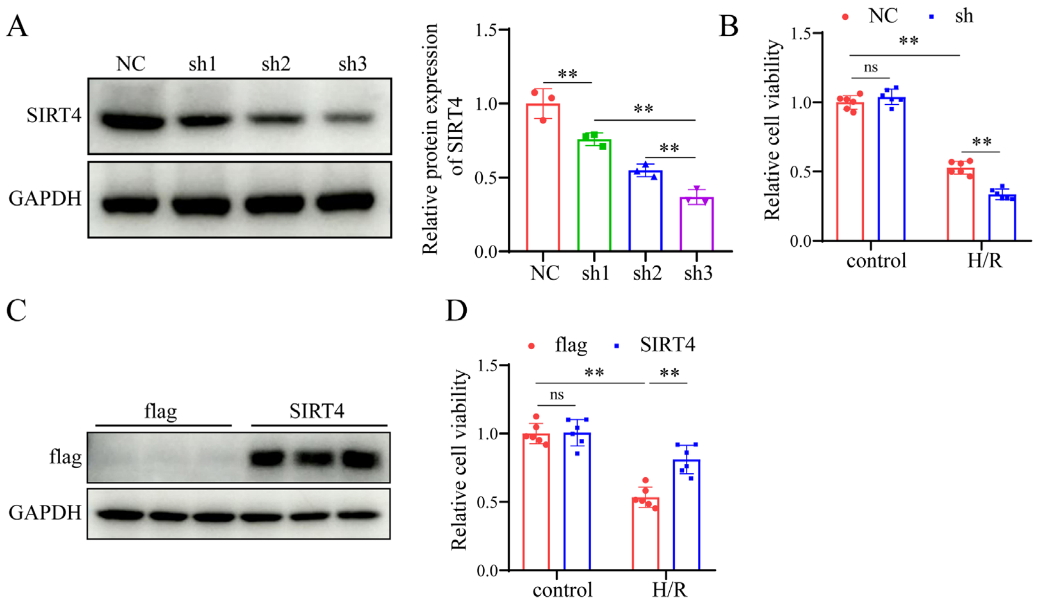

Supplementary Figure 1. SIRT4 ameliorates H/R-induced AML12 cell injury in vitro. (A) SIRT4 protein expression and statistical analysis in stable knockdown AML12 hepatocytes (n=3/group). Based on the results, the sh3 sequence was used to construct stable knockdown AML12 cells for subsequent experiments. (B) Cell activity of AML12 hepatocytes in different groups indicated 6 h reoxygenation after 12 h hypoxia (n=6/group). (C) SIRT4 protein expression in stable overexpression AML12 hepatocytes (n=3/group). (C) Cell activity of AML12 hepatocytes in different groups indicated 6 h reoxygenation after 12 h hypoxia (n=6/group). All data are presented as the mean ± SD. Levels of statistical significance are indicated as *P < 0.05, **P < 0.01.

Supplementary Figure 2

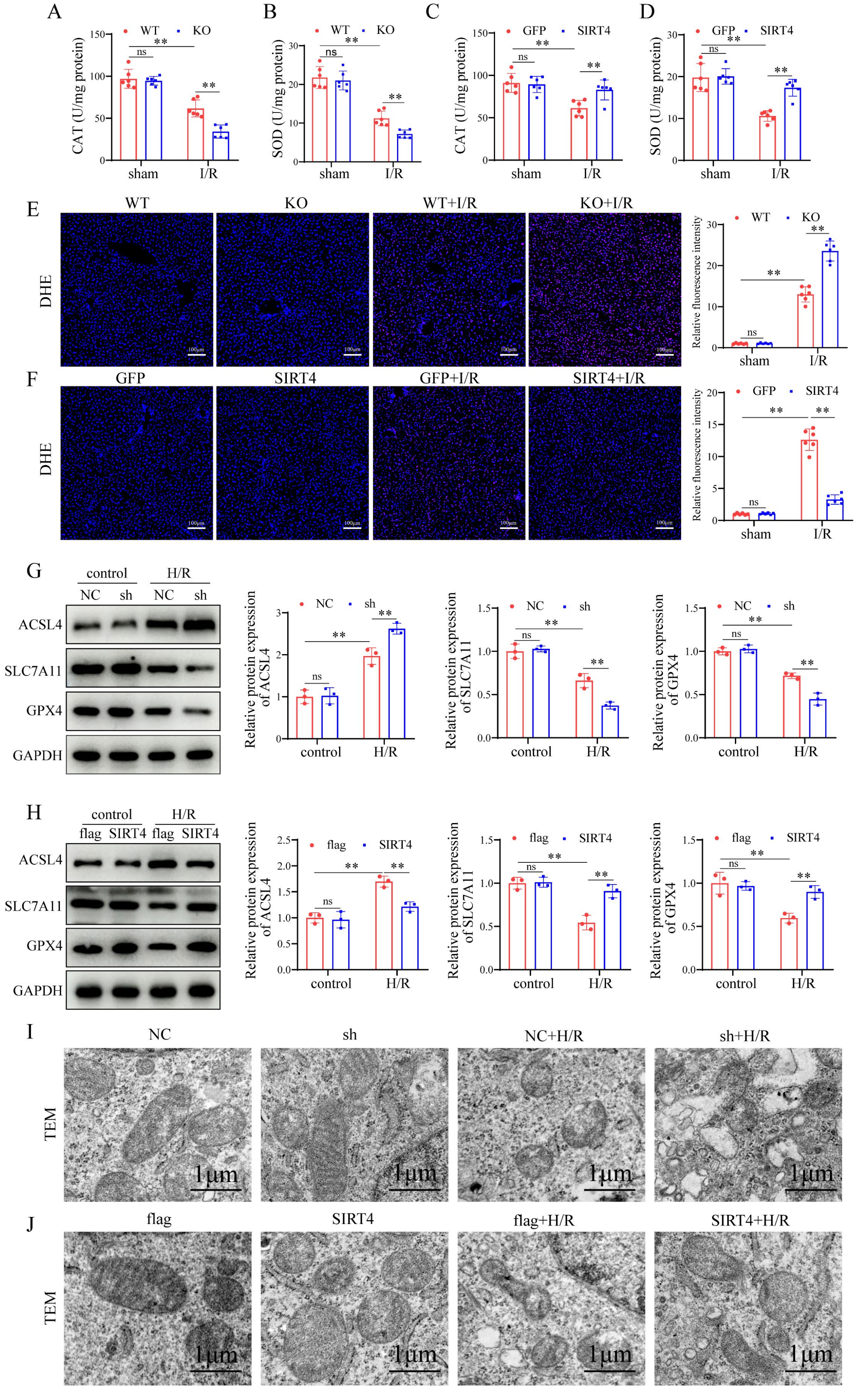

Supplementary Figure 2. SIRT4 negatively regulates ferroptosis during liver I/R injury

in vitro. (A-B) CAT and SOD levels in liver tissue from WT and KO mice under different conditions (n=6/group). (C-D) CAT and SOD levels in liver tissue from AAV-GFP and AAV-SIRT4 mice under different conditions (n=6/group). (E) DHE staining of liver tissue from WT and KO mice under different conditions (n=6/group). (F) DHE staining of liver tissue from AAV-GFP and AAV-SIRT4 mice under different conditions (n=6/group). (G) Protein detection and statistical analysis of ACSL4, SLC7A11 and GPX4 in SIRT4 knockdown and control AML12 cell with different treatment (n=3/group). (H) Protein detection and statistical analysis of ACSL4, SLC7A11 and GPX4 in SIRT4 overexpression and control AML12 cell with different treatment (n=3/group). (I) Mitochondrial structure of NC and SIRT4-sh AML12 cell with different treatment were observed by TEM. (J) Mitochondrial structure of flag and SIRT4-OE AML12 cell with different treatment were observed by TEM. All data are presented as the mean ± SD. Levels of statistical significance are indicated as *P < 0.05, **P < 0.01.

Supplementary Figure 3

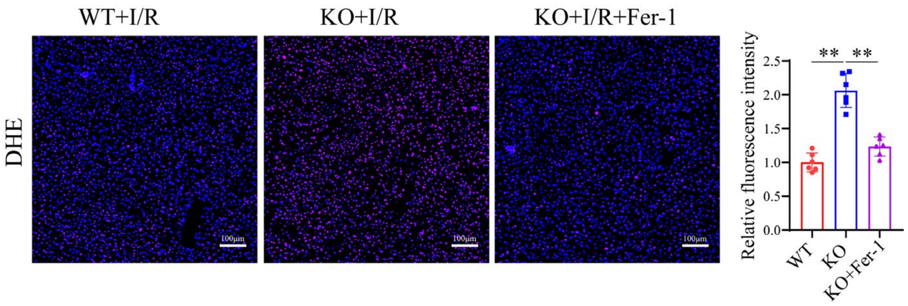

Supplementary Figure 3. SIRT4 deficiency induces liver I/R injury through ferroptosis. DHE staining of liver tissue from WT and SIRT4 KO mice subjected to different treatments (n=6/group)

Supplementary Figure 4

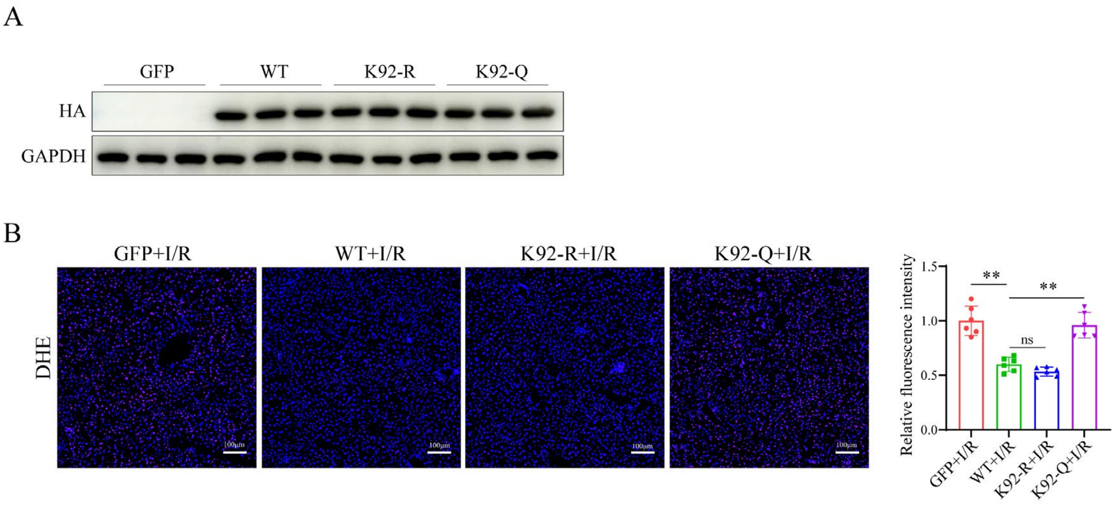

Supplementary Figure 4. Acetylation of PRDX3 at K92 negates its protective effect on LIRI. (A) Detection of PRDX3 protein in liver tissue of WT mice injected with different viruses for 4 weeks. (B)DHE staining of liver tissue from different groups of mice (n=6/group).

Supplementary Figure 5

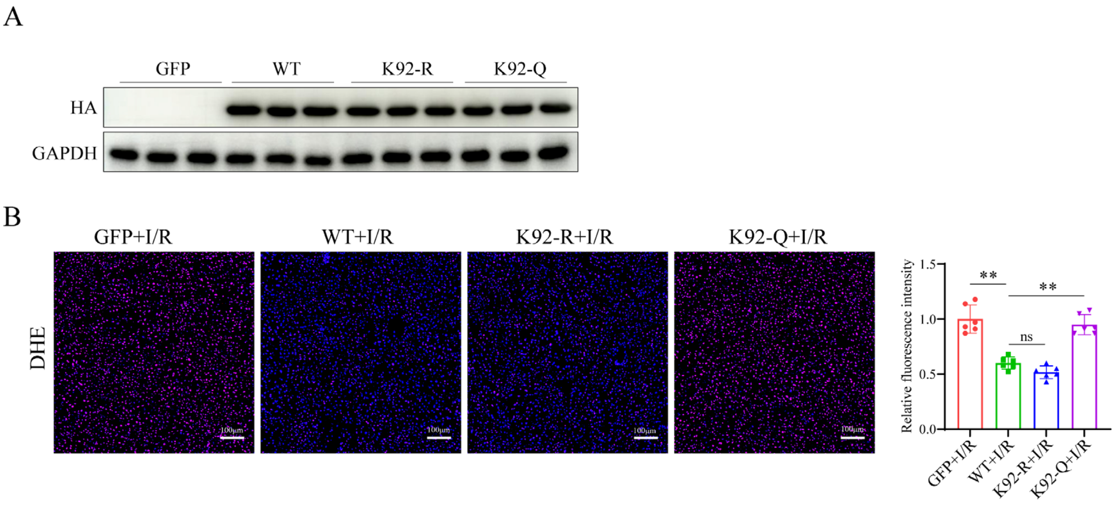

Supplementary Figure 5. The protective effect of SIRT4 against LIRI depends on the deacetylation of PRDX3 at K92. (A) Detection of PRDX3 protein in liver tissue of sirt4 KO mice injected with different viruses for 4 weeks. (B) DHE staining of liver tissue from SIRT4 KO mice subjected to different treatments (n=6/group)

Supplementary Figure 6

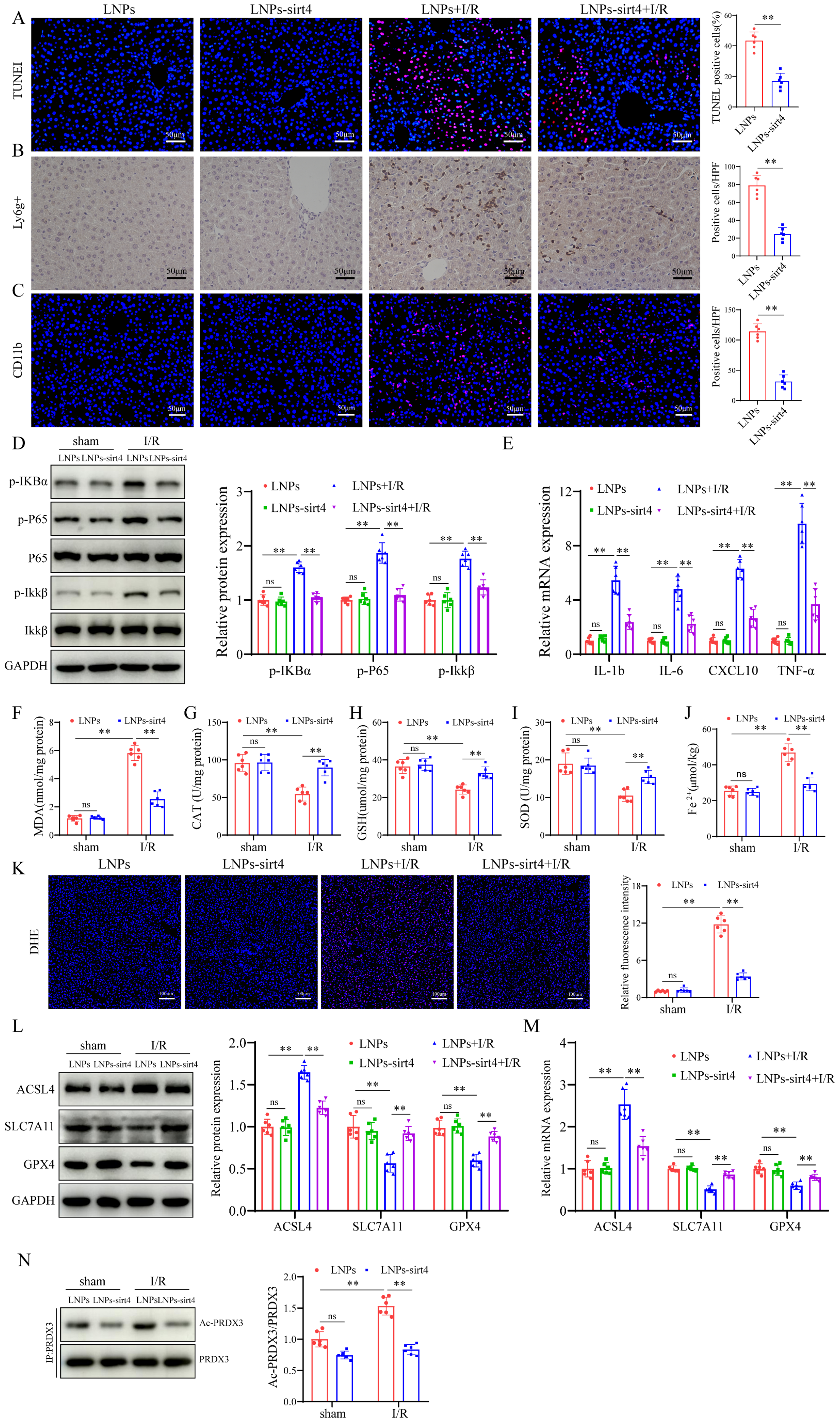

A
B
C
D
E
F
G
H
I
J
K
L
M
N
LNPs
LNPs-sirt4
LNPs+I/R
LNPs-sirt4+I/R
TUNEL
Ly6g+
CD11b
DHE
50μm
TUNEL positive cells(%)
Positive cells/HPF
sham
I/R
p-IKBα
p-P65
P65
p-Ikkβ
Ikkβ
GAPDH
Relative protein expression
Relative mRNA expression
IL-1b
IL-6
CXCL10
TNF-α
MDA(nmol/mg protein)
CAT (U/mg protein)
GSH(umol/mg protein)
SOD (U/mg protein)
Fe 2+(μmol/kg)
Relative fluorescence intensity
ACSL4
SLC7A11
GPX4
IP:PRDX3
Ac-PRDX3
PRDX3
Ac-PRDX3/PRDX3
ns
**

Supplementary Figure 6. Liver-targeted LNPs-sirt4 mRNA alleviates liver I/R injury. (A) TUNEL staining and statistical analysis of liver tissue in different groups of mice (n=6/group). (B) Immunohistochemical staining and statistical analysis of Ly6g-positive inflammatory cells in different groups of mice (n=6/group). (C) Immunofluorescence staining and statistical analysis of CD11b-positive inflammatory cells (red) in different groups of mice (n=6/group). (D) Detection of NF-κB signaling proteins and statistical analysis of liver tissue from different mice in the indicated groups (n=6/group). (E) mRNA expression of inflammatory cytokines IL-1β, IL-6, CXCL10 and TNF-α in liver tissues from different mice in the indicated groups (n=6/group). (F-I) MDA, GSH, SOD and CAT levels in liver tissue from different mice in the indicated groups (n=6/group). (J) Serum Fe^{2+} level in liver tissue from different mice in the indicated groups (n=6/group). (K) DHE staining of liver tissue from different mice in the indicated groups (n=6/group). (L) Protein detection and statistical analysis of ACSL4, SLC7A11 and GPX4 in liver tissue from different mice in the indicated groups (n=6/group). (M) mRNA expression of ACSL4, SLC7A11 and GPX4 in liver tissue from different mice in the indicated groups (n=6/group). (N) Expression and statistical analysis of acetylated PRDX3 protein in liver tissue from different mice in the indicated groups (n=6/group). All data are presented as the mean ± SD. Levels of statistical significance are indicated as *$P < 0.05$, **$P < 0.01$.

Supplementary Tables

Supplementary Table 1. Primer sequences used for RT-PCR

Primer	Primer sequence
Mouse-Sirt4 F	TAGCCGTTCACACGTAGCTG
Mouse-Sirt4 R	GGCCTGAAAGTCAATCCGCT
Mouse-IL-1β F	GCTTCAGGCAGGCAGTATCA
Mouse-IL-1β R	AGTCACAGAGGATGGGCTCT
Mouse-IL-6 F	AGAGACTTCCATCCAGTTGCC
Mouse-IL-6 R	TCCTCTGTGAAGTCTCCTCTCC
Mouse-TNF-α F	AGCCGATGGGTTGTACCTTG
Mouse-TNF-α R	ATAGCAAATCGGCTGACGGT
Mouse-CXCL10 F	ATCATCCCTGCGAGCCTATCCT
Mouse-CXCL10 R	GACCTTTTTTGGCTAAACGCTTTC
Mouse-ACSL4 F	CCTTTGGCTCATGTGCTGGAAC
Mouse-ACSL4 R	GCCATAAGTGTGGGTTTCAGTAC
Mouse-SLC7A11 F	CTTTGTTGCCCTCTCCTGCTTC
Mouse-SLC7A11 R	CAGAGGAGTGTGCTTGTGGACA
Mouse-GPX4 F	CCTCTGCTGCAAGAGCCTCCC
Mouse-GPX4 R	CTTATCCAGGCAGACCATGTGC
Mouse-GAPDH F	CTGCCCAGAACATCATCCCT
Mouse-GAPDH R	TACTTGGCAGGTTTCTCCAGG
Human-SIRT4 F	GTGGATGCTTTGCACACCAAGG
Human-SIRT4 F	GGTTCAGGACTTGGAAACGCTC
Human-GAPDH F	GTCTCCTCTGACTTCAACAGCG
Human-GAPDH F	ACCACCCTGTTGCTGTAGCCAA

Supplementary Table 2. Primary antibody information

Antibody	Supplier	Cat No.	Concentration
SIRT4	Abclonal	A7585	1:2000 for WB
p-p65	CST	3033	1:1000 for WB
p65	Proteintech	10745-1-AP	1:1000 for WB
p-IKKβ	CST	2697	1:1000 for WB
IKKβ	Proteintech	15649-1-AP	1:600 for WB
p-IkBα	CST	2859	1:1000 for WB
ACSL4	HUABIO	ET7111-43	1:1000 for WB
SLC7A11	Immunoway	YT8130	1:1000 for WB
GPX4	CST	52455	1:1000 for WB
PRDX3	Proteintech	15310-1-AP	1:5000 for WB; 2 μg for IP
Anti-Acetyllysine	PTM BIO	PTM-102	1:1000 for WB
Anti-HA Tag	HUABIO	0906-1	1:5000 for WB;2 μg for IP
Anti-flag Tag	Proteintech	80010-1-RR	1:5000 for WB;2 μg for IP
GAPDH	Proteintech	60004-1-Ig	1:5000 for WB

Supplementary Table 3. SIRT4 knockdown lentivirus plasmid sequence

plasmid	sequence
NC	GCACCTTATGCGGACGCTTTG
Sh-1	GCAGCATATCCGGAACCTTAA
Sh-2	AGTAAACCACAACTGTCTATG
sh-3	TGGAGAGTTGCTGCCTTTAAT
